# Proteome‐Wide Association Study for Finding Druggable Targets in Progression and Onset of Parkinson's Disease

**DOI:** 10.1111/cns.70294

**Published:** 2025-02-26

**Authors:** Chenhao Gao, Haobin Zhou, Weixuan Liang, Zhuofeng Wen, Wanzhe Liao, Zhixin Xie, Cailing Liao, Limin He, Jingzhang Sun, Zhilin Chen, Duopin Li, Naijun Yuan, Chuiguo Huang, Jiewen Zhang

**Affiliations:** ^1^ Department of Neurology, Henan Provincial People's Hospital Zhengzhou University People's Hospital Zhengzhou Henan China; ^2^ The First School of Clinical Medicine Guangzhou Medical University Guangzhou China; ^3^ The Sixth School of Clinical Medicine Guangzhou Medical University Guangzhou China; ^4^ Nanshan School of Guangzhou Medical University Guangzhou China; ^5^ The Second School of Clinical Medicine Guangzhou Medical University Guangzhou China; ^6^ School of Pediatrics Guangzhou Medical University Guangzhou China; ^7^ School of Cyberspace Security Hainan University Haikou China; ^8^ Department of Breast Surgery The First Affiliated Hospital of Hainan Medical University Haikou China; ^9^ The First Affiliated Hospital of Zhengzhou University Zhengzhou China; ^10^ School of Traditional Chinese Medicine Jinan University Guangzhou China; ^11^ Department of Medicine and Therapeutics, Prince of Wales Hospital The Chinese University of Hong Kong Hong Kong China

**Keywords:** causal proteins, drug repurposing, Mendelian randomization, Parkinson's disease, proteome‐wide association study, therapeutic targets

## Abstract

**Objective:**

To identify and validate causal protein targets that may serve as potential therapeutic interventions for both the onset and progression of Parkinson's disease (PD) through integrative proteomic and genetic analyses.

**Method:**

We utilized large‐scale plasma and brain protein quantitative trait loci (pQTL) datasets from the deCODE Health study and the Religious Orders Study/Rush Memory and Aging Project (ROS/MAP), respectively. Proteome‐wide association studies (PWAS) were conducted using the OTTERS framework for plasma proteins and the FUSION tool for brain proteins, examining associations with PD onset and three progression phenotypes: composite, motor, and cognitive. Significant protein associations (FDR‐corrected *p* < 0.05) from PWAS were further validated using summary‐based Mendelian randomization (SMR), colocalization analyses, and reverse Mendelian randomization (MR) to establish causality. Phenome‐wide Mendelian randomization (PheW‐MR) was performed to assess potential side effects across 679 disease traits when targeting these proteins to reduce PD‐related phenotype risk by 20%. Additionally, we conducted cellular distribution‐based clustering using gene expression data from the Allen Brain Atlas (ABA) to explore the distribution of key proteins across brain regions, constructed protein–protein interaction (PPI) networks via the STRING database to explore interactions among proteins, and evaluated the druggability of identified targets using the DrugBank database to identify opportunities for drug repurposing.

**Result:**

Our analyses identified 25 candidate proteins associated with PD phenotypes, including 16 plasma proteins linked to PD progression (10 cognitive, 4 motor, and 3 composite) and 9 plasma proteins associated with PD onset. Notably, GPNMB was implicated in both plasma and brain tissues for PD onset. PheW‐MR revealed predominantly beneficial side effects for the identified targets, with 83.7% of associations indicating positive outcomes and 16.3% indicating adverse effects. Cellular clustering categorized candidate targets into three distinct expression profiles across brain cell types using ABA. PPI network analysis highlighted one key interaction cluster among the proteins for PD cognitive progression and PD onset. Druggability assessment revealed 15 out of 25 proteins had repurposing opportunities for PD treatment.

**Conclusion:**

We have identified 25 causal protein targets associated with the onset and progression of PD, providing new insights into the research and development of treatment strategies for PD.

## Graphical abstract

We identified and validated 25 causal protein targets linked to Parkinson's disease (PD) onset and progression using proteomic and genetic analyses. Using plasma and brain large‐scale pQTL datasets, we conducted proteome‐wide association studies (PWAS) and Mendelian randomization (MR) to assess causality. Our findings revealed 16 plasma proteins associated with PD progression and 9 with PD onset. Notably, GPNMB was implicated in both. Our study provides new insights for PD therapeutic strategies.

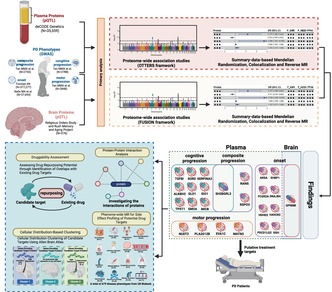

**Abbreviations**
ABA: Allen Brain Atlas
ACAT‐O: Aggregated Cauchy Association Test
BH: Benjamini–Hochberg
FDR: false discovery rate
FUSION: Functional Summary‐based Imputation
GreX: genetically regulated expression
GWAS: genome‐wide association studies
HEIDI: Heterogeneity in Dependent Instruments
IVs: instrumental variables
Lassosum: a frequentist LASSO‐based approach
LD: linkage disequilibrium
NSAIDs: nonsteroidal anti‐inflammatory drugs
OTTERS: Omnibus Transcriptome Test using Expression Reference Summary data
PD: Parkinson's Disease
PheW‐MR: phenome‐wide MR
PP: posterior probability
PPI: protein–protein interaction
pQTL: protein quantitative trait loci
PRS‐CS: a Bayesian multivariable regression model utilizing continuous shrinkage priors
P+T: *p*‐value thresholding with LD clumping
PWAS: proteome‐wide association studies
ROS/MAP: Religious Orders Study/Rush Memory and Aging Project
SDPR: a nonparametric Bayesian Dirichlet Process Regression model
SMR: summary‐based Mendelian randomization
SNPs: single nucleotide polymorphisms
STRING: Search Tool for the Retrieval of Interacting Genes/Proteins
UPGMA: Unweighted Pair Group Method with Arithmetic Mean

## 1 Introduction

Parkinson's disease (PD) is a neurodegenerative disorder characterized by the progressive loss of dopaminergic neurons in the substantia nigra pars compacta and the accumulation of α‐synuclein aggregates, known as Lewy bodies. It is the second most prevalent neurodegenerative disease after Alzheimer's disease [1, 2]. Epidemiological studies indicate a global increase in PD cases, rising from 2.5 million to 6.1 million over the past three decades [3]. With the aging global population, the incidence of PD is projected to escalate significantly, imposing substantial socioeconomic burdens on patients and healthcare systems [4].

PD manifests through a spectrum of motor symptoms, including tremors, rigidity, bradykinesia, and postural instability, resulting from the degeneration of dopaminergic neurons [1, 2, 5, 6]. In addition to these motor deficits, PD encompasses a range of non‐motor symptoms such as cognitive decline, mood disorders, and autonomic dysfunction, which contribute to the disease's complexity and severely impact the quality of life of affected individuals [7]. The heterogeneity in disease progression, characterized by varying rates of motor and cognitive deterioration among patients, presents significant challenges for effective treatment and management strategies. Current therapeutic approaches for PD primarily aim at symptomatic relief, employing medications like levodopa and dopamine agonists to replenish dopamine levels and alleviate motor symptoms [8]. While these treatments can provide temporary improvement, they do not halt the underlying neurodegenerative processes driving the disease [8]. The absence of disease‐modifying therapies underscores the urgent need for interventions that can influence both the onset and progression of PD.

Advancements in proteomics and genomics have opened new avenues for identifying biomarkers and therapeutic targets in complex diseases such as PD. Proteome‐wide association studies (PWAS), leveraging protein quantitative trait loci (pQTL) data, facilitate the identification of protein‐level associations with disease phenotypes [9]. Specifically, plasma and brain proteomics offer valuable insights into systemic and central nervous system‐specific protein alterations linked to PD [10, 11]. Integrative genomic analyses that combine genome‐wide association studies (GWAS) with proteomic data enable the elucidation of causal relationships between genetic variants, protein expression, and disease traits [10, 11]. Methodologies such as PWAS, summary‐based Mendelian randomization (SMR) [12], colocalization analyses [13], and phenome‐wide MR (PheW‐MR) [14] are instrumental in dissecting the genetic architecture of PD and identifying proteins that may serve as potential therapeutic targets. Therefore, by integrating these powerful and steady approaches in a logical order, our study aims to identify latent but reliable drug targets for PD.

**FIGURE 1:**
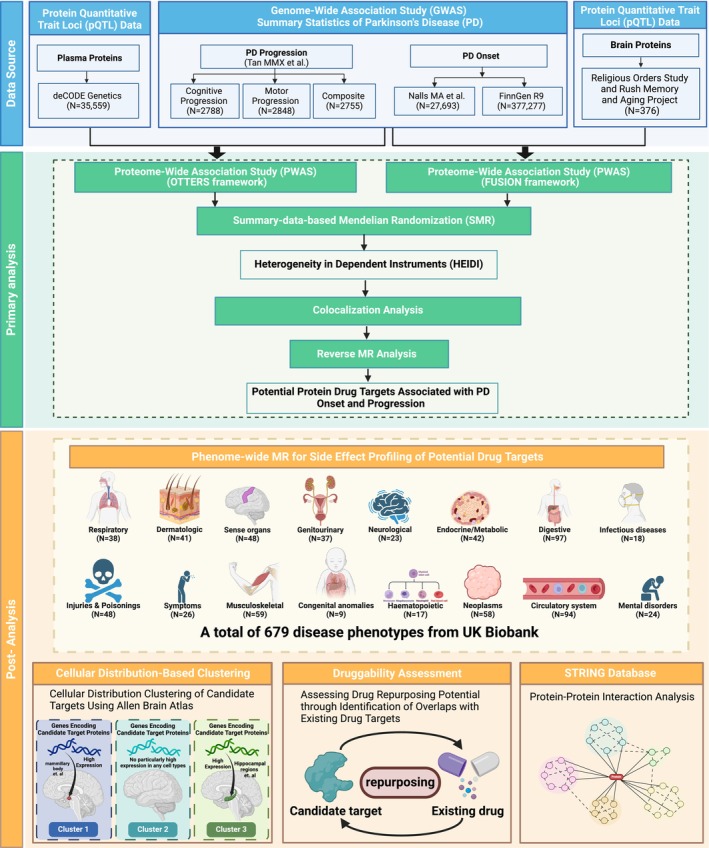
Flowchart of the study design.

Few studies that explored PD's targets focused on the developing procedure of this neurodegenerative disease, while the primary objective of our study is to identify and validate potential therapeutic targets for both the onset and progression of PD through integrative proteomic and genetic analyses, providing novel perspectives on the dynamic changes associated with PD. By harnessing large‐scale plasma and brain pQTL datasets from the deCODE Health study and the Religious Orders Study/Rush Memory and Aging Project (ROS/MAP), respectively, we conducted comprehensive PWAS to uncover proteins associated with various PD phenotypes, including PD onset and three distinct progression phenotypes: composite, motor, and cognitive. These PD phenotypes were selected to comprehensively capture the entire disease trajectory. Subsequent sensitivity analyses, including SMR and colocalization, were employed to confirm the causal relevance of these proteins and PD phenotypes. Additionally, a reverse MR analysis was performed to explore potential bidirectional causal relationships between proteins and PD. The identification of causal proteins may highlight candidate drug targets for PD treatment. Furthermore, PheW‐MR analyses were conducted to assess potential side effects of targeting these candidate proteins, thereby informing the safety and efficacy of prospective therapeutic interventions for PD. Given that PD primarily affects the brain, it is essential to understand the cellular distribution of the genes encoding candidate drug target proteins across various brain regions to develop effective therapies. To achieve this, we utilized gene expression data from the ABA to perform cluster analysis, refining the distribution of candidate targets within the brain and identifying co‐expression patterns in specific cell populations. Moreover, we employed protein–protein interaction (PPI) networks to investigate interactions between candidate proteins across multiple PD phenotypes, thereby elucidating functional relationships and exploring the potential for multi‐target drug development. Then, we integrated drug target information from the DrugBank database to explore opportunities for drug repurposing of candidate targets [15]. Figure 1 shows the research workflow of this study. Taken together, the identification of causal protein targets not only enhances our understanding of PD pathogenesis but also paves the way for the development of disease‐modifying therapies and personalized medicine approaches aimed at improving patient outcomes.

## 2 Method

### 2.1 Data Sources

We obtained plasma pQTL data from the deCODE Health study, which performed comprehensive proteomic profiling in plasma samples from 35,559 Icelandic participants using the SomaScan platform, ultimately quantifying 4907 distinct plasma proteins [16]. For brain‐derived protein data, we used pQTL information on 1097 proteins measured in the dorsolateral prefrontal cortex from participants in the ROS/MAP using mass spectrometry [17]. We also incorporated GWAS summary statistics for three PD progression phenotypes, including composite (2755 patients), motor (2848 patients), and cognitive (2788 patients), as reported by Tan MMX et al. [18] For PD onset, the discovery cohort consisted of GWAS summary statistics derived from Nalls MA et al. [19] (15,056 cases and 12,637 controls), and the replication cohort employed data from the FinnGen consortium [20] (4235 cases and 373,042 controls). Details of these datasets are provided in Table S1.

### 2.2 PWAS

We conducted PWAS on both brain and whole blood tissues to identify protein‐level associations with PD phenotypes. For brain tissue, we utilized the Functional Summary‐based Imputation (FUSION) framework, which employs existing pQTL weights specifically tailored to brain proteomes [21]. FUSION is a well‐established computational tool that imputes genetically regulated gene expression and assesses gene‐level associations with complex traits and diseases. By leveraging pretrained pQTL weights for brain tissue, we integrated PD‐related phenotypes and performed PWAS using FUSION on a Linux platform [22].

In contrast, appropriate pretrained PWAS weights for whole blood were unavailable. To overcome this limitation, we employed the Omnibus Transcriptome Test using Expression Reference Summary data (OTTERS), a specialized framework designed to generate and utilize pQTL weights from summary‐level data [23]. OTTERS operates in two primary stages. In Stage I, we constructed genetically regulated expression (GReX) imputation models by deriving cis‐pQTL weights, defined as the regions extending 1 MB upstream and downstream of the protein‐coding genes, from summary‐level cis‐pQTL data and external European linkage disequilibrium (LD) reference panels from the 1000 Genomes Project. Multiple methodologies were employed for weight derivation, including P+T (*p*‐value thresholding with LD clumping) [24], lassosum (a frequentist LASSO‐based approach) [25], SDPR (a nonparametric Bayesian Dirichlet Process Regression model) [26, 27], and PRS‐CS (a Bayesian multivariable regression model utilizing continuous shrinkage priors) [28]. In Stage II, these cis‐pQTL weights were used to estimate GReX for each gene, enabling gene‐level association tests within the GWAS dataset. PWAS *p*‐values derived from each modeling approach were subsequently integrated into a single composite metric using the Aggregated Cauchy Association Test (ACAT‐O) [29]. We refer to the resultant *p*‐values from this integrated test as OTTERS *p*‐values.

For our analyses, we incorporated plasma pQTL data from the deCODE Health Study and brain pQTL data from the Religious Orders Study and the Rush Memory and Aging Project (ROS/MAP). We applied the Benjamini–Hochberg (BH) method to correct *p*‐values and control the false discovery rate (FDR), thereby minimizing false positives without excessively inflating false negatives. In the PWAS, proteins with FDR‐corrected *p*‐values below 0.05 were considered significantly associated with the corresponding PD phenotype. Specifically, for proteins associated with PD onset, those that reached significance in the discovery cohort and maintained *p* < 0.05 in the replication cohort were deemed successfully replicated and selected for subsequent analyses.

### 2.3 Sensitivity Analyses

#### 2.3.1 SMR Analysis

To rigorously validate our PWAS findings, we employed SMR to confirm both brain and plasma proteins found to be causally associated with PD‐related phenotypes. SMR integrates pQTL and GWAS summary statistics within the MR framework, which utilizes instrumental variables (IVs), genetic variants that serve as proxies for protein levels, to enable the assessment of the causal impact of protein levels on PD‐related traits [12]. SMR is an extension of MR, and MR adheres to three core assumptions: (i) the relevance assumption, which requires a strong association between IVs and the exposure; (ii) the independence assumption, stating that IVs influence the outcome solely through the exposure; (iii) the exclusion restriction assumption, which dictates that IVs should not have a direct impact on the outcome. Unlike conventional two‐sample MR, where two independent GWAS datasets are required to estimate the causal effect between traits, SMR combines pQTL and GWAS data and utilizes the Heterogeneity in Dependent Instruments (HEIDI) test [30]. This approach offers more robust discrimination between pleiotropic and linkage effects, reduces potential biases due to LD, and lowers the large sample size requirements often seen in standard MR methods [30].

We adopted the SMR‐derived estimates as our primary measures of each protein's influence on PD‐related phenotypes. Given the inherent stringency of the SMR method, we applied the Benjamini–Hochberg procedure to control the FDR, thereby minimizing false positives without excessively inflating false negatives. Any protein that met the criteria of an FDR‐adjusted SMR *p* < 0.05 and a HEIDI *p* > 0.01 was considered to have a causal relationship with the respective PD‐related phenotype [31].

The threshold for the *p*‐value of the IVs was 5e‐08 when running SMR. To ensure the robustness of our IVs, we calculated *F*‐statistics using the established formula [32]:

$$F=\frac{r^{2}\left(N-2\right)}{\left(1-r^{2}\right)}$$

where *N* is the sample size and *r* ^2^ is the proportion of variance in the exposure explained by the IV. An *F*‐statistic greater than 10 is commonly regarded as indicative of sufficient IV strength, thus mitigating weak instrument bias. All calculated *F*‐values are presented in Table S2.

#### 2.3.2 Colocalization Analysis

To determine whether the observed associations between proteins and PD‐related phenotypes stemmed from a shared causal variant rather than LD, we conducted Bayesian colocalization analyses using the coloc R package [13]. This methodology integrated both brain and plasma pQTL data with GWAS summary statistics for PD‐related traits. We evaluated five distinct hypotheses: (i) H0: no causal variant influences either the protein or PD‐related phenotypes; (ii) H1: a causal variant affects only the protein; (iii) H2: a causal variant affects only the PD phenotype; (iv) H3: distinct causal variants influence the protein and PD phenotypes independently; and (v) H4: a single causal variant affects both. For each protein, we included single nucleotide polymorphisms (SNPs) within a ± 500 kb window surrounding its pQTL region. In instances where a protein was associated with multiple pQTLs, each pQTL was analyzed separately, prioritizing those with the strongest evidence of association. A posterior probability (PP) greater than 0.8 for hypothesis H4 was considered strong evidence supporting the existence of a shared causal variant. Overall, the prioritized proteins, which were significantly identified in PWAS and had successfully passed SMR, HEIDI, and colocalization assessments, might have the potential to be the candidate targets for PD treatment.

#### 2.3.3 Reverse MR Analysis

Complementing our primary SMR and colocalization analyses, we implemented a reverse MR approach to investigate potential bidirectional causal relationships among candidate targets [33, 34]. In this analysis, GWAS data for PD‐related phenotypes were designated as exposures, while proteins that satisfied the PWAS, SMR, HEIDI, and colocalization thresholds from both whole‐blood and brain pQTL datasets were treated as outcomes. To ensure an adequate number of IVs for each PD‐related phenotype, we adopted a relaxed significance threshold of *p* < 5e‐06 and performed LD clumping to maintain LD independence (*r* ^2^ < 0.001, window size = 10,000 kb) among the SNPs.

Subsequently, we calculated *F*‐statistics for each IV to assess their strength, excluding those with *F*‐values < 10 to mitigate weak instrument bias. Following this, the Steiger test was conducted to verify the directionality of the associations, retaining only SNPs that explained a larger proportion of variance in the exposure compared to the outcome (Table S3). This step ensured that each IV primarily influenced the outcome through its effect on the exposure. Finally, we applied a Bonferroni‐corrected significance threshold of *p* < (0.05/*n*), where n denotes the total number of the tested associations, with associations surpassing this threshold considered statistically significant, thereby revealing potential bidirectional causalities between PD and the candidates.

### 2.4 PheW‐MR Analysis of 679 Disease Traits

To evaluate the potential unintended consequences of targeting proteins implicated in PD‐related phenotypes, we conducted a PheW‐MR analysis encompassing 679 distinct disease traits. Initially, we established causal associations between our prioritized proteins and these 679 common disease traits using PheW‐MR. Subsequently, we integrated these results with the SMR findings for PD‐related phenotypes to ensure that potential side effects were not confounded by directional biases. This comprehensive approach enabled the identification of unintended consequences associated with targeting specific proteins as therapeutic interventions.

For this analysis, protein–disease associations were derived from PheW‐MR evaluations across a broad spectrum of 679 diseases, each comprising more than 500 cases, as previously described by Zhou et al. [14] These phenotypes were sourced from the UK Biobank (*N* ≤ 408,961) and categorized using PheCodes. To determine the effect sizes of proteins on the 679 diseases, we performed MR. In this process, IVs were selected using a stringent significance threshold of *p* < 5e‐08, followed by LD clumping (*r* ^2^ < 0.1, window size = 10,000 kb) to ensure LD independence among the selected SNPs.

The effects of proteins on PD‐related phenotypes were obtained from SMR analyses linking the candidate proteins to PD‐related traits. We defined a side effect as any influence on an alternate disease trait resulting from manipulating a target protein to achieve a 20% reduction in the risk of the PD‐related phenotype. To estimate and standardize the magnitude of side effects, we adopted the following formula for the odds ratio of the effect (OR_effect_) [35]:

$${\text{OR}}_{\text{effect}}=\exp\left(\beta_{\text{effect}}\right)$$

where

$$\beta_{\text{effect}}=\frac{\beta_{679\;\text{diseases}}}{\beta_{\mathrm{PD}\;\text{phenotypes}}}\times \ln\left(0.8\right)$$

Here, $\beta_{679\;\text{diseases}}$ represents the effect of the candidate proteins on the 679 diseases, with only associations having *p*‐values below 0.05 included. $\beta_{\mathrm{PD}\;\text{phenotypes}}$ denotes the proteins' effect on PD‐related phenotypes, derived from the PheW‐MR and SMR analyses. Proteins with OR values greater than 1 were considered to have potentially adverse side effects, whereas those with OR values less than 1 were deemed to confer beneficial side effects. *p*‐values were estimated using a bootstrap method with 1 million iterations (*n* = 1,000,000) and the *p*‐values for the side effects were corrected using the Bonferroni method. A side effect was considered statistically significant if its *p*‐value was below (0.05/*k*). Here, *k* represents the total number of associations between proteins and diseases that had a *p* < 0.05 in the PheW‐MR analysis.

The associations achieving Bonferroni‐corrected significance threshold of *p*‐values < (0.05/*k*), where *k* is the total number of protein–disease associations identified at *p* < 0.05, were regarded as statistically significant [36].

### 2.5 Cellular Distribution‐Based Clustering of Candidate Targets Using ABA Data

Given that PD predominantly affects the brain, it is imperative to elucidate the cellular distribution of genes encoding candidate target proteins across various brain regions to develop effective therapies. To refine the spatial distribution of these targets and identify co‐expression patterns within specific cell populations, we conducted cluster analysis using gene expression data from the ABA [37]. Specifically, we utilized the Whole Human Brain 10x RNA‐seq dataset (data updated on March 30, 2024) and extracted log₂‐normalized expression matrices corresponding to our prioritized protein‐coding genes. Cluster analysis was performed based on the similarity of gene expression levels across different cell types. Hierarchical clustering was executed using the Unweighted Pair Group Method with Arithmetic Mean (UPGMA) to identify patterns of co‐expression and potential functional relationships among the genes.

### 2.6 PPI Network and Druggability Assessment

To identify synergistic interactions among targets across multiple phenotypes and facilitate the development of multi‐target therapeutics, we constructed a PPI network encompassing candidate targets associated with various PD phenotypes. We utilized the Search Tool for the Retrieval of Interacting Genes/Proteins (STRING) database (version 12.0; http://string‐db.org) to identify interactions among proteins implicated in both the onset and progression of PD, as determined in our preceding analyses. An interaction score threshold of ≥ 0.4 was applied to ensure a moderate level of confidence in the identified interactions. The resulting PPI network was subsequently visualized using Cytoscape (version 3.6.1; https://cytoscape.org). For clarity, any nodes not connected to the main PPI network were excluded from the final visualization.

To evaluate the feasibility of drug repurposing, we conducted a druggability assessment using real‐world drug target data from databases such as DrugBank. This assessment enabled us to identify overlaps between our identified proteins and established drug targets, as well as to explore their associated therapeutic indications. By leveraging preprocessed data from Ruiz et al. [38], we facilitated the evaluation of potential repurposing opportunities, thereby enhancing the clinical relevance of our candidate proteins for the treatment of Parkinson's disease.

## 3 Result

### 3.1 PWAS for PD Progression and Onset

#### 3.1.1 Identification of Plasma Proteins Associated With PD Progression

**FIGURE 2:**
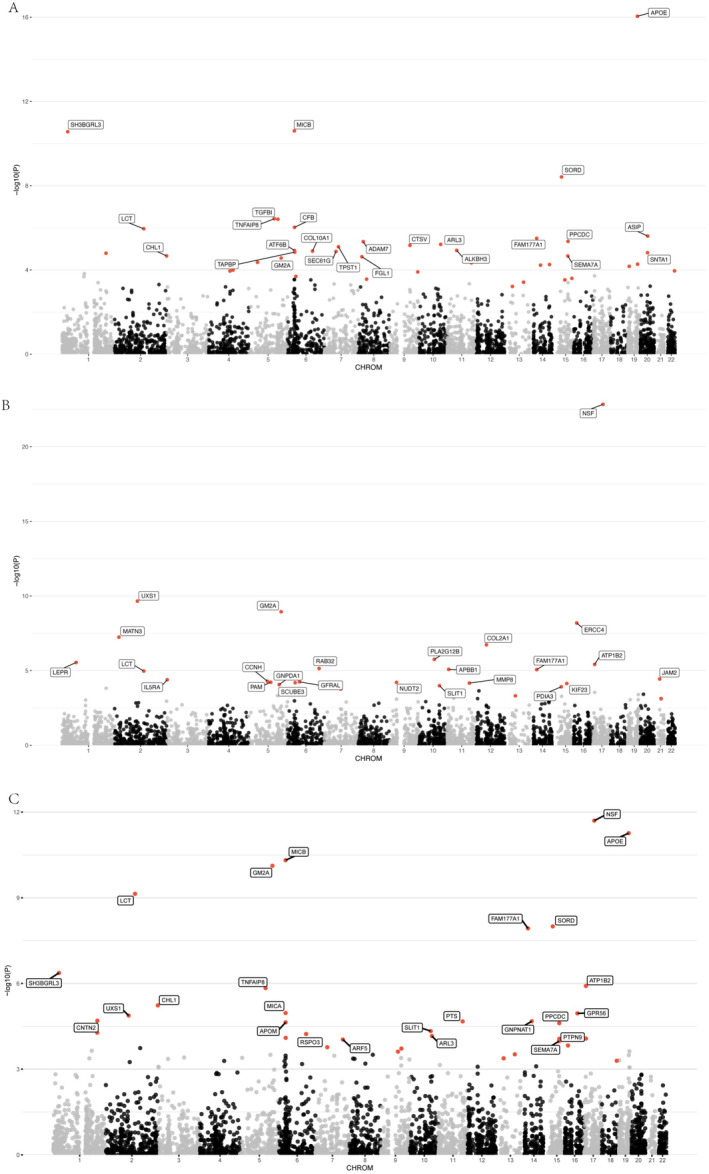
Manhattan plots showing PWAS analysis results of plasma proteins associated with PD progressions. Proteins significantly associated with PD progressions are marked with red dots (PBonferroni < 0.05) in the Manhattan plots. (A) Manhattan plot of PWAS analysis for plasma proteins and cognitive progression in PD. (B) Manhattan plot of PWAS analysis for plasma proteins and motor progression in PD. (C) Manhattan plot of PWAS analysis for plasma proteins and composite progression in PD.

We integrated plasma pQTL data from the deCODE study with GWAS summary statistics for three PD statuses (cognitive, motor, and composite progression) and conducted a PWAS using the OTTERS framework, encompassing 4732 proteins. Proteins were deemed significantly associated with PD progression if they met the FDR‐corrected significance threshold (*p* < 0.05). Our analysis identified 42 plasma proteins associated with cognitive progression, 30 with motor progression, and 39 with composite progression (see Table S4 and Figure 2A–C). Notably, APOE exhibited the most significant association with cognitive progression (*p* = 3.12e‐14), while NSF was most significantly associated with both motor (*p* = 3.90e‐21) and composite (*p* = 5.42e‐10) progressions (Table S4).

**FIGURE 3:**
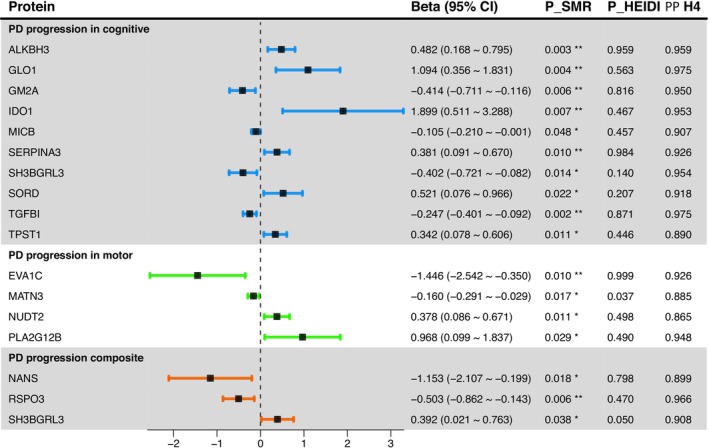
Forest plot showing plasma proteins validated to be causally associated with PD progression in cognitive, motor, and composite phenotypes. The plot displays effect estimates (*β* values) and significance levels (*p*‐values) from SMR and HEIDI analyses, together with posterior probabilities (PP_H4_) from colocalization analysis.

To validate the causal associations between plasma proteins and the PD progression phenotypes, we performed SMR and HEIDI analyses. Applying stringent criteria—SMR *p* (FDR‐corrected) < 0.05 and HEIDI *p* > 0.05—we identified 12 plasma proteins causally associated with cognitive progression, 5 with motor progression, and 6 with composite progression (Table S5, Figure 3). A subsequent colocalization analysis (PP_H4_ > 0.8) confirmed that, of the 12 proteins linked to cognitive progression, 10 (ALKBH3, GLO1, IDO1, SERPINA3, SORD, TPST1, GM2A, MICB, SH3BGRL3, and TGFBI) shared causal variants with PD cognitive progression loci (Table S6, Figure 3). Among these 10 proteins, the abundance of ALKBH3 (*β* = 0.482, *p* = 2.58e‐03), GLO1 (*β* = 1.094, *p* = 3.64e‐03), IDO1 (*β* = 1.899, *p* = 7.34e‐03), SERPINA3 (*β* = 0.381, *p* = 9.87e‐03), SORD (*β* = 0.521, *p* = 2.17e‐02), and TPST1 (*β* = 0.342, *p* = 1.10e‐02) exhibited significant positive causal correlations with cognitive progression, whereas GM2A (*β* = −0.414, *p* = 6.48e‐03), MICB (*β* = −0.105, *p* = 4.79e‐02), SH3BGRL3 (*β* = −0.402, *p* = 1.36e‐02), and TGFBI (*β* = −0.247, *p* = 1.76e‐03) demonstrated negative correlations. Of the five proteins associated with motor progression, four (NUDT2, PLA2G12B, EVA1C, and MATN3) passed the colocalization test. Specifically, the abundance of NUDT2 (*β* = 0.378, *p* = 1.11e‐02) and PLA2G12B (*β* = 0.968, *p* = 2.90e‐02) correlated positively with motor progression, whereas EVA1C (*β* = −1.446, *p* = 9.73e‐03) and MATN3 (*β* = −0.160, *p* = 1.69e‐02) correlated negatively. Additionally, among the six proteins implicated in composite progression, three (SH3BGRL3, NANS, and RSPO3) were validated by colocalization analysis. SH3BGRL3 (*β* = 0.392, *p* = 3.84e‐02) was positively correlated with composite progression, while NANS (*β* = −1.153, *p* = 1.78e‐02) and RSPO3 (*β* = −0.503, *p* = 6.19e‐03) exhibited negative correlations. Notably, SH3BGRL3 emerged as a causal factor in both cognitive and composite progressions, despite manifesting a negative correlation with the former and a positive correlation with the latter. Finally, reverse MR analyses revealed no significant bidirectional associations (*p* < 0.05/114) between these proteins and the three PD progression phenotypes (Table S7).

#### 3.1.2 Identified Related Plasma Proteins for the Onset of PD

**FIGURE 4:**
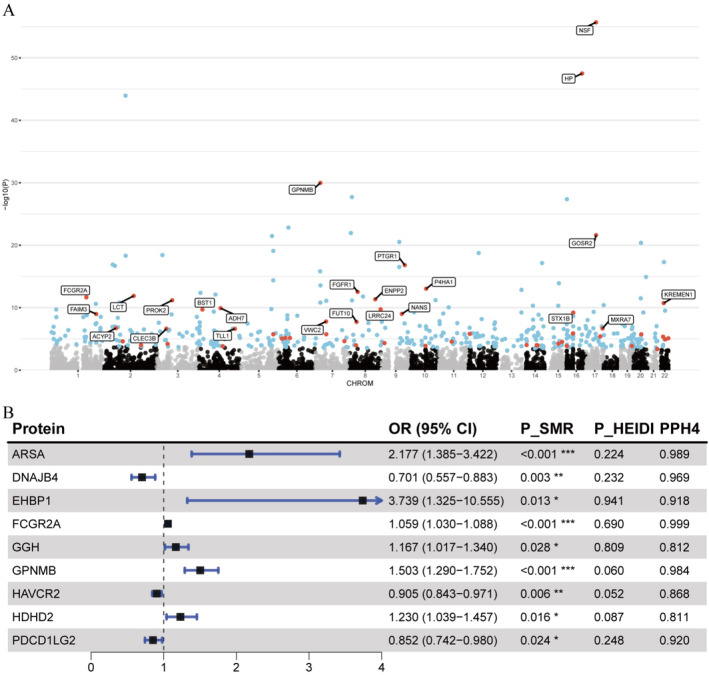
Manhattan plot and forest plot showing plasma proteins associated with PD onset. (A) Manhattan plot of PWAS findings in discovery and replication cohorts. Proteins that were significant in the discovery cohort and successfully replicated in the replication cohort (*p*_discovery_ < 0.05 and *p*_replication_ < 0.05) are represented by red dots; proteins that were significant only in the discovery cohort but did not successfully replicate in the replication cohort (*p*_discovery_ < 0.05 and *p*_replication_ > 0.05) are shown as blue dots. (B) Forest plot of plasma proteins associated with PD onset. The plot shows OR values and *p*‐values from SMR and HEIDI analyses, along with the PP_H4_ from colocalization analysis.

Using the OTTERS framework, we conducted a PWAS on 4693 proteins in both the discovery and replication datasets to identify plasma proteins linked to PD onset. Proteins meeting the FDR‐corrected significance threshold (*p* < 0.05) were considered significantly associated with PD onset. In the discovery dataset, we identified 317 proteins correlated with PD onset, while 230 proteins were implicated in the replication dataset. Notably, 54 proteins reached significance in both datasets, with NSF exhibiting the most robust association (*p*_discovery_ = 2.06e‐56, *p*_replication_ = 1.87e‐224) (Table S4, Figure 4A). To further evaluate their potential causal roles in PD onset, the 54 proteins were subjected to subsequent sensitivity analyses.

We applied SMR and the HEIDI test to these 54 proteins to explore their causal associations with PD onset. Overall, 22 plasma proteins demonstrated significant causal evidence for PD onset (*P*_*SMR (FDR‐corrected*)_ < 0.05 and *P*_*HEIDI*_ > 0.01) (Table S5). Colocalization analysis confirmed that nine of these proteins (ARSA, EHBP1, FCGR2A, GGH, GPNMB, HDHD2, DNAJB4, HAVCR2, and PDCD1LG2) shared a common causal variant with PD onset (PP_H4_ > 0.8; Table S6, Figure 4B). Among these nine proteins, the abundance of six proteins, ARSA (OR = 2.177,*p* = 7.53e‐04), EHBP1 (OR = 3.739, *p* = 1.27e‐02), FCGR2A (OR = 1.059, *p* = 4.37e‐05), GGH (OR = 1.167, *p* = 2.81e‐02), GPNMB (OR = 1.503, *p* = 1.79e‐07), and HDHD2 (OR = 1.230, *p* = 1.64e‐02), was significantly associated with an elevated risk of PD onset, whereas the abundance of DNAJB4 (OR = 0.701, *p* = 2.54e‐03), HAVCR2 (OR = 0.905, *p* = 5.55e‐03), and PDCD1LG2 (OR = 0.852, *p* = 2.44e‐02) was associated with a reduced risk. To examine potential bidirectional causal relationships, we also performed reverse MR analyses with no significant association detected, reinforcing the robustness of the observed causal links between the identified proteins and PD onset (Table S7).

#### 3.1.3 Identified Related Proteins With Human Brain Proteomes for the Progression of PD

We utilized the FUSION framework to perform PWAS analyses on brain pQTLs, evaluating the associations between 1097 proteins and the progression of PD. Our analysis identified 57 proteins associated with cognitive progression, 45 with motor progression, and 55 with composite progression (*p* < 0.05). Among these, MICAL1 exhibited the strongest associations with both cognitive progression (*p* = 1.38e‐03) and composite progression (*p* = 1.36e‐03), while C14orf159 was most significantly associated with motor progression (*p* = 2.54e‐03). Despite these findings, no proteins reached the FDR‐corrected significance threshold (*p* < 0.05) for any of the PD progression phenotypes. Consequently, no brain proteins were identified as candidate targets for further sensitivity analyses (Figures S1–S3 and Table S8).

#### 3.1.4 Identified Related Proteins With Human Brain Proteomes for the PD Onset

**FIGURE 5:**
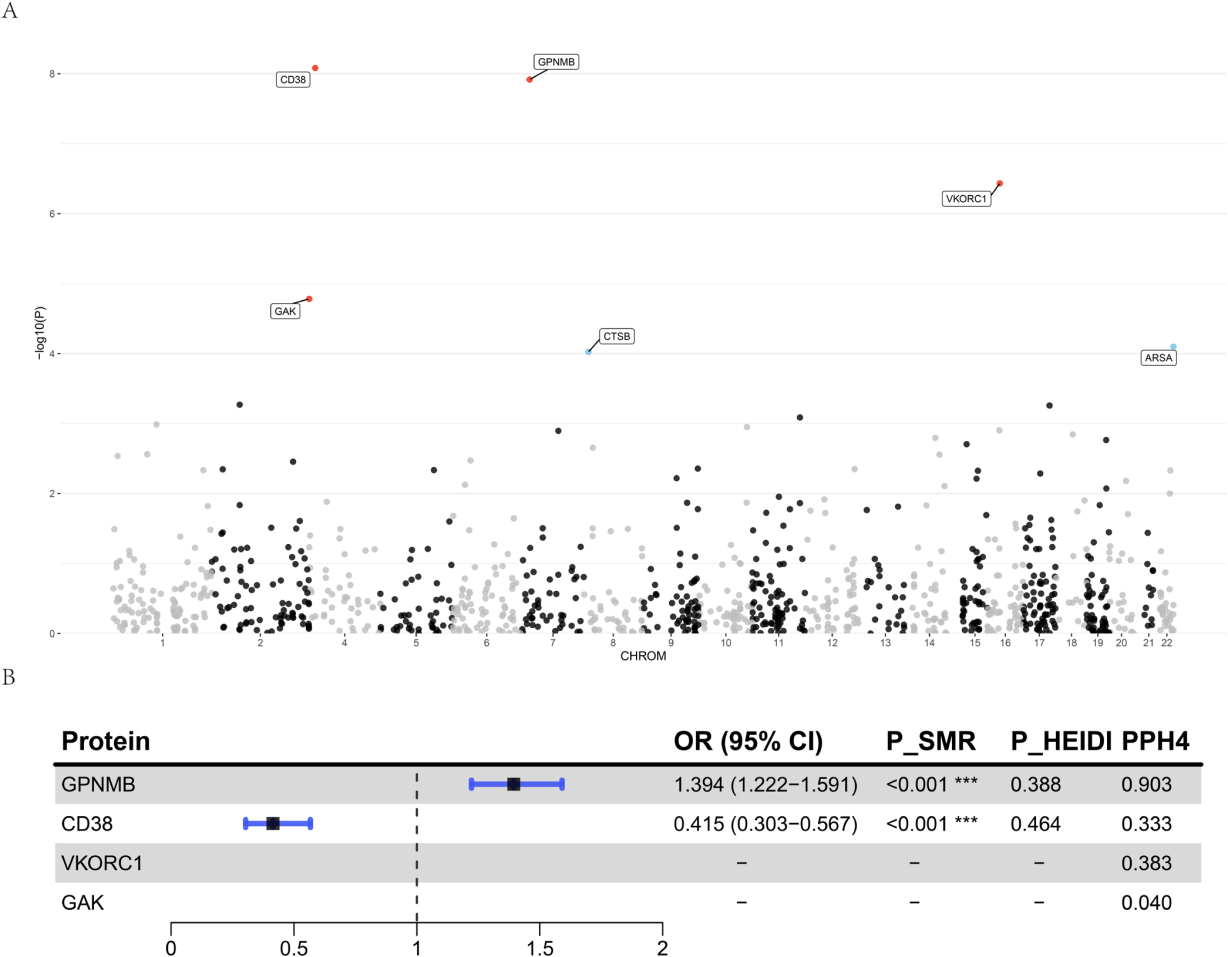
Manhattan plot and forest plot showing brain proteins associated with PD onset. (A) Manhattan plot of PWAS findings in discovery and replication cohorts. Proteins that were significant in the discovery cohort and successfully replicated in the replication cohort (*p*_discovery_ < 0.05 and *p*_replication_ < 0.05) are represented by red dots; proteins that were significant only in the discovery cohort but did not successfully replicate in the replication cohort (*p*_discovery_ < 0.05 and *p*_replication_ > 0.05) are shown as blue dots. (B) Forest plot of brain proteins associated with PD onset. The plot shows OR values and *p*‐values from SMR and HEIDI analyses, along with the PP_H4_ from colocalization analysis.

We employed the FUSION framework for PWAS analysis to leverage brain pQTL data in assessing the association between 1067 proteins and the onset of PD (Figure 5, Table S8). In the discovery cohort, 99 proteins demonstrated significant associations with PD onset (*p* < 0.05). After applying the FDR correction, four proteins remained significantly associated and were subsequently validated in replication cohorts (*p* < 0.05). These proteins include CD38 (*p*_discovery_ = 8.27e‐09, *p*_replication_ = 0.004), GPNMB (*p*_discovery_ = 1.21e‐08, *p*_replication_ = 0.034), VKORC1 (*p*_discovery_ = 1.65e‐05, *p*_replication_ = 0.015), and GAK (*p*_discovery_ = 3.69e‐07, *p*_replication_ = 0.003). Additionally, CTSB (*p*_discovery_ = 9.47 × 10^−5^, *p*_replication_ = 0.477) and ARSA (*p*_discovery_ = 7.95 × 10^−5^, *p*_replication_ = 0.153) were found to be significantly associated with PD onset in the discovery cohort but did not reach the *p* < 0.05 threshold in the replication cohort.

For the four proteins validated through PWAS, we initially performed SMR and the HEIDI test to elucidate their causal relationships with PD onset (Table S5). Among these four proteins, only GPNMB and CD38 had valid IVs extracted from brain pQTL data. Consequently, we conducted SMR and HEIDI tests exclusively for these two proteins. The results revealed that both GPNMB and CD38 exhibited significant causal associations with PD onset. Specifically, the abundance of GPNMB (OR = 1.394, *p* = 7.73e‐07) was associated with an increased risk of PD onset, whereas the abundance of CD38 (OR = 0.415, *p* = 3.32e‐08) was associated with a decreased risk. Subsequent colocalization analysis using the COLOC method confirmed the associations between these two proteins and PD onset (Table S6). The analysis showed that only GPNMB had PP.H4 exceeding 0.8, indicating a shared causal variant between GPNMB and PD onset. As a final sensitivity analysis, we attempted to perform a reverse MR analysis to investigate the association between GPNMB and PD onset. Unfortunately, because we only had SNPs within the GPNMB cis region, and there was no overlap with the IVs for PD onset, we did not have valid IVs for the analysis, thereby precluding the reverse MR analysis for this protein. This limitation prevents us from fully establishing the bidirectional causal relationship of this protein, necessitating further investigation in future studies.

#### 3.1.5 Summary of Candidate Plasma and Brain Targets Related to PD Phenotypes

Our analyses identified 25 candidate targets associated with PD‐related phenotypes. Among these, 16 plasma proteins were linked to PD progression. Specifically, 10 plasma proteins (ALKBH3, GLO1, IDO1, SERPINA3, SORD, TPST1, GM2A, MICB, SH3BGRL3, and TGFBI) exhibited causal relationships with cognitive progression, four proteins (NUDT2, PLA2G12B, EVA1C, and MATN3) were associated with motor progression, and three proteins (SH3BGRL3, NANS, and RSPO3) were linked to composite progression. Notably, SH3BGRL3 emerged as a causal factor for both cognitive and composite progressions. Additionally, nine plasma proteins (ARSA, EHBP1, FCGR2A, GGH, GPNMB, HDHD2, DNAJB4, HAVCR2, and PDCD1LG2) demonstrated causal relationships with PD onset.

**FIGURE 6:**
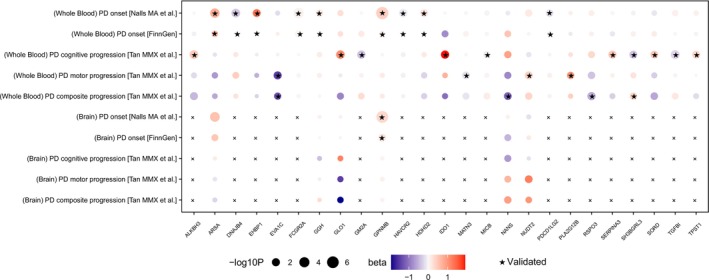
Bubble plot summarizing candidate proteins identified from the integrated analyses. Circle colors indicate effect sizes (*β* values) from SMR analysis, while circle sizes represent −log10(*p*‐values). Asterisk (*) indicates proteins that were significantly identified by PWAS and validated through SMR, HEIDI, COLOC, and reverse MR analyses, and “x” indicates no results.

When applying the same analytical pipeline to brain proteins, we did not identify any brain‐specific candidate targets causally linked to PD progression. However, we identified one protein in brain tissue, GPNMB, as a candidate target showing a clear causal association with PD onset. Intriguingly, GPNMB was implicated in PD onset in both plasma and brain tissues. These results are summarized in Figure 6 and Table 1.

**TABLE 1:** Descriptive summary of 25 potential target proteins of the study analyses.

Source	Target	PD‐related phenotype	P_PWAS_	P_SMR_	PP.H4
Plasma	ALKBH3	Cognitive progression	1.17e‐05	0.003	0.959
Plasma	GLO1	Cognitive progression	2.02e‐04	0.004	0.975
Plasma	GM2A	Cognitive progression	2.70e‐05	0.006	0.950
Plasma	IDO1	Cognitive progression	2.75e‐04	0.007	0.953
Plasma	MICB	Cognitive progression	2.49e‐11	0.048	0.907
Plasma	SERPINA3	Cognitive progression	5.53e‐05	0.010	0.926
Plasma	SH3BGRL3	Cognitive progression	2.73e‐11	0.014	0.954
Plasma	SORD	Cognitive progression	3.83e‐09	0.022	0.918
Plasma	TGFBI	Cognitive progression	3.90e‐07	0.002	0.975
Plasma	TPST1	Cognitive progression	7.91e‐06	0.011	0.890
Plasma	EVA1C	Motor progression	7.63e‐04	0.010	0.926
Plasma	MATN3	Motor progression	5.69e‐08	0.017	0.885
Plasma	NUDT2	Motor progression	6.13e‐05	0.011	0.865
Plasma	PLA2G12B	Motor progression	1.75e‐06	0.029	0.948
Plasma	NANS	Composite progression	1.90e‐04	0.018	0.899
Plasma	RSPO3	Composite progression	5.87e‐05	0.006	0.966
Plasma	SH3BGRL3	Composite progression	4.27e‐07	0.038	0.908
Plasma	ARSA	Onset	1.58e‐06	< 0.001	0.989
Plasma	DNAJB4	Onset	3.99e‐07	0.003	0.969
Plasma	EHBP1	Onset	1.34e‐07	0.013	0.918
Plasma	FCGR2A	Onset	2.19e‐12	0.013	0.918
Plasma	GGH	Onset	1.63e‐12	< 0.001	0.812
Plasma	GPNMB	Onset	1.04e‐30	0.028	0.984
Plasma	HAVCR2	Onset	4.86e‐10	< 0.001	0.868
Plasma	HDHD2	Onset	1.40e‐06	0.006	0.811
Plasma	PDCD1LG2	Onset	1.80e‐08	0.015	0.920
Brain	GPNMB	Onset	1.21e‐08	< 0.001	0.903

*Note:* This table reported candidate drug target source, PD‐related phenotypes, P_PWAS_, P_SMR_, and PP.H4 for each protein.

Abbreviations: ALKBH3, alpha‐ketoglutarate‐dependent dioxygenase AlkB homolog 3; ARSA, arylsulfatase A; DNAJB4, DnaJ homolog subfamily B member 4; EHBP1, EH domain binding protein 1; EVA1C, protein Eva‐1 homolog C; FCGR2A, low affinity immunoglobulin gamma Fc region receptor II‐a; GGH, gamma‐glutamyl hydrolase; GLO1, lactoylglutathione lyase; GM2A, ganglioside GM2 activator; GPNMB, transmembrane glycoprotein NMB; HAVCR2, hepatitis A virus cellular receptor 2; HDHD2, haloacid dehalogenase‐like hydrolase domain‐containing protein 2; IDO1, indoleamine 2,3‐dioxygenase 1; MATN3, matrilin‐3; MICB, MHC class I polypeptide‐related sequence B; NANS, sialic acid synthase; NUDT2, Bis(5′‐nucleosyl)‐tetraphosphatase [asymmetrical]; PDCD1LG2, programmed cell death 1 ligand 2; PLA2G12B, group XIIB secretory phospholipase A2‐like protein; PP.H4, *p*‐value of colocalization; P_PWAS_, *p*‐value of PWAS; P_SMR_, *p*‐value of SMR; RSPO3, R‐spondin‐3; SERPINA3, alpha‐1‐antichymotrypsin; SH3BGRL3, SH3 domain‐binding glutamic acid‐rich‐like protein 3; SORD, sorbitol dehydrogenase; TGFBI, transforming growth factor‐beta‐induced protein ig‐h3; TPST1, indicates protein‐tyrosine sulfotransferase 1.

### 3.2 PheW‐MR

Following the identification of candidate targets associated with PD‐related phenotypes, we conducted a comprehensive analysis across 679 common disease traits to characterize the side effect profiles of each prioritized protein as a potential therapeutic target. Unlike the previous SMR approach, PheW‐MR results were standardized to reflect a 20% reduction in the risk of PD‐related phenotypes mediated by each protein. Consequently, the observed associations can be interpreted as potential side effects that may arise from therapeutically targeting these proteins.

**FIGURE 7:**
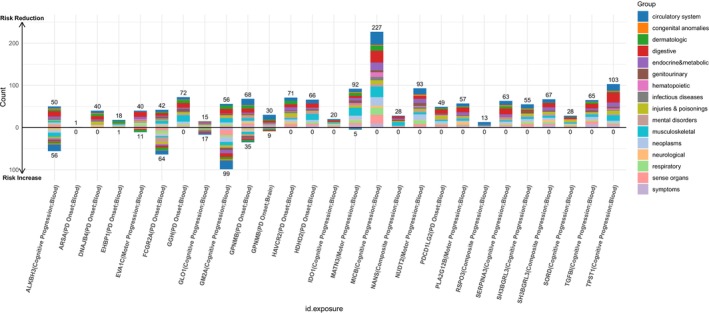
Bar plot representing PheW‐MR analysis results between candidate protein targets and 679 disease traits. Each protein target is represented by a set of bars. Different colors indicate distinct disease systems. The x‐axis shows individual candidate proteins, while the y‐axis presents the direction of effect: Upward bars indicate beneficial associations (risk reduction) when targeting the protein, and downward bars indicate adverse associations (risk increase). The height of each bar represents the number of associations.

Under this 20% risk‐reduction assumption, PheW‐MR analyses (*p* < 0.05/3126) identified 1529 significant beneficial side effects (83.7%) and 297 adverse side effects (16.3%) across 25 candidate targets. A paired t‐test confirmed that beneficial side effects significantly outnumbered adverse side effects (*p* = 7.91e‐05), suggesting that the majority of identified side effects were beneficial (Table S9, Figure 7). Of these 25 candidate targets, 17 exhibited exclusively beneficial side effects, while the remaining eight displayed both beneficial and adverse side effects.

Among the 17 candidate targets with exclusively beneficial side effects, we focused on those that reduce the risk of four major PD progression phenotypes and demonstrated the largest number of positive outcomes. For targets mitigating PD cognitive progression, MICB exhibited the most pronounced beneficial profile, with 227 beneficial side effects primarily concentrated in the circulatory system (31 distinct traits). Regarding PD motor progression, NUDT2 was associated with 93 beneficial side effects, predominantly within the circulatory system (15 traits). For PD composite progression, SH3BGRL3 conferred 67 beneficial side effects—the highest in this category—primarily related to digestive disorders. Finally, among candidate targets for PD onset, GGH showed 72 beneficial side effects, mainly affecting musculoskeletal conditions.

In contrast, the remaining eight candidate targets displayed both beneficial and adverse side effects. Notably, targeting PD cognitive progression, GM2A was linked to 155 total side effects, comprising 56 beneficial and 99 adverse effects. For PD motor progression, EVA1C yielded 51 side effects, including 40 beneficial and 11 adverse effects. Lastly, for PD onset, FCGR2A was associated with 106 side effects, including 42 beneficial and 64 adverse effects. No significant side effects were detected among the candidate targets for PD composite progression.

### 3.3 Cellular Distribution‐Based Clustering of Genes Corresponding to Candidate Targets

To elucidate the cellular distribution of genes encoding candidate target proteins across various brain regions for the development of effective PD therapies, we retrieved gene expression matrices from the ABA covering 31 distinct brain cell types. Of the 25 proteins identified, we successfully obtained corresponding gene expression data for 24, excluding EHBP1, which lacked expression information. We then performed hierarchical clustering using the Unweighted Pair Group Method with Arithmetic Mean (UPGMA) on these 24 genes based on their expression patterns across the 31 cell types.

The clustering analysis resulted in three distinct clusters. Cluster 1, comprising solely TPST1, exhibited elevated expression primarily in deep‐layer intratelencephalic and near‐projecting neurons, as well as in the mammillary body and the lower rhombic lip. Cluster 2 included GPNMB, SORD, GM2A, PDCD1LG2, MATN3, TGFBI, FCGR2A, DNAJB4, MICB, SERPINA3, IDO1, and PLA2G12B, none of which showed particularly high expression in any of the examined cell types. Cluster 3 consisted of EVA1C, GLO1, SH3BGRL3, RSPO3, GGH, NANS, ARSA, NUDT2, HAVCR2, ALKBH3, and HDHD2, all demonstrating elevated expression in metabolic and homeostatic cell populations, notably within hippocampal regions (CA1–CA3, CA4, and the dentate gyrus), deep‐layer corticothalamic neurons, and vascular cells. Detailed results of the clustering analysis are provided in Table S10.

### 3.4 PPI Network

**FIGURE 8:**
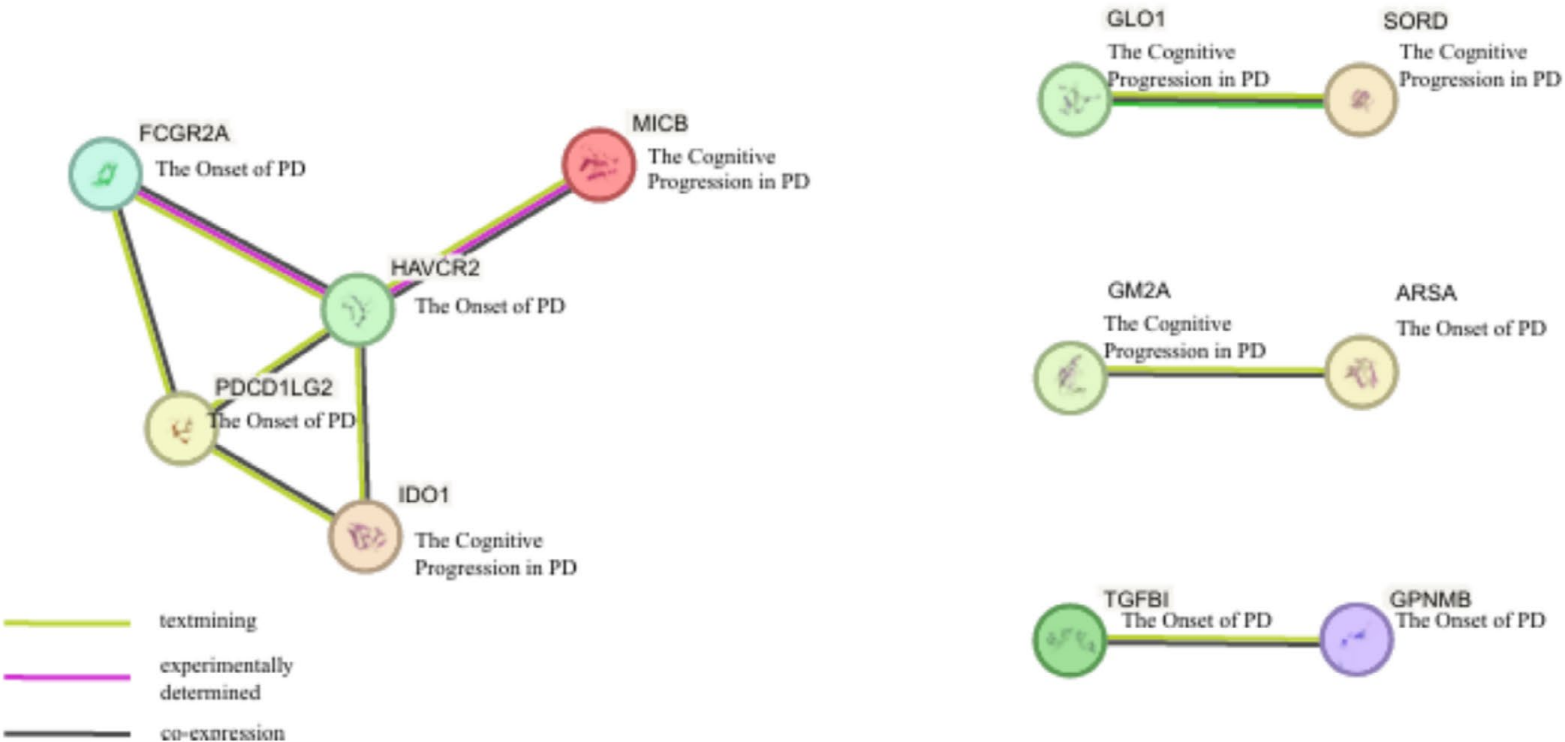
PPI network constructed using STRING database to identify interactions between candidate proteins involved in PD progression and onset. Different colored lines connecting proteins indicate the sources of identified protein–protein interactions: Black lines represent direct interaction/co‐expression, green lines denote text‐mining evidence, and purple lines indicate experimentally determined relationships.

To elucidate synergistic relationships among targets across diverse PD phenotypes, we examined interactions among the 25 candidate proteins using a PPI network constructed via the STRING database (Figure 8). The PPI network analysis identified a primary interaction cluster comprising FCGR2A, HAVCR2, PDCD1LG2, and IDO1, which were interconnected with MICB. Specifically, FCGR2A, HAVCR2, and PDCD1LG2 were associated with PD onset, whereas MICB and IDO1 were linked to cognitive progression in PD. Additionally, multiple pairwise interactions were observed. GLO1 and SORD, both associated with cognitive progression, formed a direct interaction pair. GM2A, also related to cognitive progression, interacted with ARSA, a candidate target for PD onset. Furthermore, our PPI analysis revealed an interaction between TGFBI and GPNMB, both of which have been implicated in PD onset.

### 3.5 Druggability Assessment

To explore the potential for repurposing existing medications targeting the candidate proteins, we consulted the DrugBank database to identify drugs known to modulate these targets. Of the 25 proteins identified in this study, 15 correspond to established drug targets, indicating significant overlaps with treatments for various neurological and psychiatric disorders (Table S11). Notably, EHBP1, SERPINA3, FCGR2A, GPNMB, MICB, RSPO3, NUDT2, and GLO1 are primarily associated with antipsychotic agents such as chlorpromazine, risperidone, and olanzapine. Additionally, IDO1 and GLO1 are targets for a range of antidepressants, including citalopram, fluoxetine, and venlafaxine. Furthermore, FCGR2A, NANS, and MATN3 are linked to corticosteroids and nonsteroidal anti‐inflammatory drugs (NSAIDs) like prednisone, ibuprofen, and naproxen. GGH and MATN3 are involved in pathways targeted by antiepileptic drugs, such as phenytoin and topiramate. GM2A is associated with both antiepileptic and sedative medications, while ARSA is implicated in treatments for dystonia and epilepsy.

## 4 Discussion

Our study identified and validated latent plasma and brain targets for the onset and progression of PD by an integrative PWAS, which is an effective method in such contexts. Based on extensive pQTL data from plasma and brain tissues, comprehensive GWAS summary statistics were also utilized, resulting in the identification of 25 protein targets associated with the PD trajectory, including its onset and progression. Furthermore, we provided comprehensive insights into the therapeutic potential and safety profiles for the prioritized targets through PheW‐MR analysis, cellular distribution‐based clustering, PPI networks, and druggability assessments.

Moreover, we reviewed the prioritized protein targets by accessing literature sources (including PubMed, Embase, and Google Scholar, etc.), and ALKBH3, GLO1, GM2A, IDO1, SERPINA3, TGFBI578PL, A2G12B, ARSA, FCGR2A, and GPNMB were found to be previously reported with reliable evidence [39, 40, 41, 42, 43, 44, 45, 46, 47], while MICB, SH3BGRL3, SORD, TPST1, EVA1C, MATN3, NUDT2, NANS, RSPO3, DNAJB4, EHBP1, GGH, HAVCR2, HDHD2, and PDCD1LG2 were novelly identified with few direct evidence from prior studies. Though it is relatively unreliable to predict most novel targets' concrete mechanisms in PD onset or progression, we focus on the novelty and investigability, from proteins to pathways, then to the phenotype and subtype.

Concerning the identified progression‐related targets, ALKBH3, GLO1, IDO1, SERPINA3, SORD, TPST1, GM2A, MICB, SH3BGRL3, and TGFBI, we found them to be significantly associated with cognitive decline in the plasma of PD patients. They are involved in diverse biological processes: Expression and glycation damage of GLO1 was demonstrated to be induced by alpha‐synuclein ablation, contributing to the development of PD^40^; IDO1 inhibition improves motor dysfunction and provides neuroprotection in PD mice [42]; MICB and TPST1 are novel targets identified for PD, may shape neuroinflammatory processes by modulating microglial activation in PD and mediate sulfation of key neuronal proteins modulating intracellular signaling pathways, thereby influencing dopaminergic neuron survival and accelerate the progression [48, 49]; Additionally, targets such as NUDT2, PLA2G12B, EVA1C, and MATN3 were associated with motor progression, highlighting potential targets for mitigating motor dysfunction in PD. For instance, NUDT2, involved in nucleotide metabolism [50], and PLAG12B in PLA2 (phospholipase A2) superfamily were suggestively associated with PD, influencing neuronal membrane integrity and essential signal transduction pathways for motor function [45]. The identification of these targets underscores the complex interplay between metabolic and inflammatory pathways in PD motor symptoms. Furthermore, SH3BGRL3 was identified as an intriguing target, demonstrating a dual role by being causally linked to both cognitive and composite progression phenotypes, albeit with contrasting directions of effect. While there is no direct evidence, this duality suggests that SH3BGRL3 may regulate multiple pathways that differentially affect various aspects of disease progression, such as influencing PD by stabilizing synaptic architecture and acting as a redox sensor, thereby protecting against α‐synuclein‐induced synaptic deficits and adjusting dysregulated intracellular signaling cascades [51]. Aside from that, previously hinted by an MR study, GPNMB stood out as a pivotal target showing a causal relationship with increased risk of PD onset in both plasma and brain tissues [47]. The consistent association of GPNMB across different tissues highlights its potential as a robust biomarker for early PD detection and as a promising therapeutic target to delay disease onset.

The PheW‐MR analysis offered a comprehensive evaluation of the potential side effect profiles associated with the candidate targets. Impressively, 83.7% of the identified side effects were beneficial, while 16.3% were adverse. This predominance of beneficial side effects suggests that targeting these proteins may confer therapeutic advantages beyond PD, thereby enhancing the overall safety and efficacy of potential interventions. For instance, MICB's association with numerous beneficial traits within the circulatory system underscores its potential role in vascular health, which could be advantageous given the emerging evidence of vascular contributions to PD pathology [52]. Additionally, targets such as NUDT2 and SH3BGRL3 exhibited substantial beneficial effects across various disease traits, highlighting their multifaceted therapeutic potential. Conversely, targets like GM2A and FCGR2A, which demonstrated both beneficial and adverse side effects, emphasize the necessity for cautious therapeutic modulation to balance efficacy with safety.

We also conducted Cellular Distribution‐Based Clustering to elucidate the cellular distribution of genes encoding candidate target proteins across various brain regions, thereby informing the development of effective PD therapies. This analysis identified three distinct clusters, with particular emphasis on Cluster 1 and Cluster 3. Cluster 1, comprising solely TPST1, exhibited elevated expression in deep‐layer intratelencephalic and near‐projecting neurons, as well as in the mammillary body and lower rhombic lip. This specific expression profile suggests that TPST1 may play a critical role in neuronal connectivity and signaling pathways pertinent to PD onset, presenting a targeted opportunity for therapeutic intervention. Cluster 3, consisting of EVA1C, GLO1, SH3BGRL3, RSPO3, GGH, NANS, ARSA, NUDT2, HAVCR2, ALKBH3, and HDHD2, demonstrated elevated expression in metabolic and homeostatic cell populations, particularly within hippocampal regions, deep‐layer corticothalamic neurons, and vascular cells. The metabolic and homeostatic functions highlighted by Cluster 3 underscore the importance of maintaining cellular energy balance and vascular integrity in mitigating PD‐related neurodegeneration. These findings suggest that targeting metabolic pathways and supporting vascular health could be pivotal in slowing disease progression and enhancing neuronal survival. Furthermore, we conducted PPI analysis to explore synergistic relationships among targets across diverse PD phenotypes. Utilizing the STRING database for PPI network analysis, we identified a primary interaction cluster comprising FCGR2A, HAVCR2, PDCD1LG2, and IDO1, interconnected with MICB. Notably, MICB and IDO1 emerged as candidate targets associated with PD cognitive progression, while the remaining proteins were linked to PD onset. IDO1 plays a crucial role in regulating immune responses and inflammation, potentially contributing to cognitive deterioration in PD patients [53], whereas MICB modulates natural killer and T cell activity, suggesting a complex immune regulatory mechanism underlying cognitive impairments [54]. Conversely, FCGR2A, HAVCR2, and PDCD1LG2 are primarily associated with PD onset, involving immune regulation and sustained inflammatory responses that may drive neurodegenerative changes [55, 56, 57]. This cluster highlights candidate targets associated with PD onset and cognitive progression, suggesting that targeting these interconnected proteins could modulate both the initiation and advancement of the disease. The intricate interactions among these candidate targets reveal potential nodes for multi‐target drug development, where simultaneous modulation of interconnected proteins may enhance therapeutic efficacy and more effectively mitigate disease progression compared to single‐target approaches.

Utilizing the DrugBank database, we assessed the druggability and therapeutic potential of the 25 identified candidate targets [15]. Notably, 15 of these candidates were recognized as existing drug targets, highlighting significant opportunities for drug repurposing. Proteins such as EHBP1, SERPINA3, and GLO1 are currently targeted by antipsychotic and antidepressant medications, whereas FCGR2A and MATN3 are associated with corticosteroids and NSAIDs. This overlap indicates that existing pharmacological agents could be repurposed to modulate these proteins, thereby potentially accelerating the development of disease‐modifying therapies for PD.

Our study is underpinned by several notable strengths that collectively enhance its scientific rigor and potential impact. Firstly, our research represents the first known PWAS utilizing the OTTERS method and large‐scale summary‐level pQTL data from deCODE to investigate both the onset and progression of PD. In contrast to previous PWAS studies that employed small‐sample pQTL data, we leveraged the OTTERS framework with extensive summary‐level pQTL data from deCODE. This methodological approach significantly increases statistical power, enabling the identification of a greater number of critical proteins, particularly those previously undiscovered. Consequently, this not only deepens our understanding of the mechanisms driving PD progression but also provides additional potential targets for developing disease‐modifying therapeutic strategies. Furthermore, the robustness of our findings is reinforced through rigorous validation methodologies, including SMR, colocalization analyses, and bidirectional MR. These approaches collectively ensure high confidence in the causal relationships between proteins and PD phenotypes. Additionally, our comprehensive assessment of potential side effects via PheW‐MR offers critical insights into the safety profiles of candidate targets, thereby informing the development of safer therapeutic interventions. The identification of existing drug targets among the candidates also facilitates drug repurposing, potentially accelerating the translation of our findings into clinical applications and enhancing the feasibility of novel therapeutic strategies.

Despite the comprehensive nature of this PWAS, several limitations warrant consideration. First, our analyses do not encompass the entirety of the human proteome. Some proteins remain unmeasured and may also play pivotal roles in PD onset and progression, introducing the possibility of horizontal pleiotropy. Second, although our investigation incorporated both plasma and brain pQTL datasets, the absence of training sets in OTTERS for PWAS among brain proteins may reflect limitations in assay sensitivity and completeness of the entire study pipeline, partially weakening the convincement. Third, our study primarily includes individuals of Icelandic and European ancestries, where population homogeneity might reduce the generalizability of our findings to more diverse ethnic backgrounds, underscoring the need for replication efforts in multiethnic cohorts. Fourth, different proteomic platforms were employed (SOMAscan for plasma vs. mass spectrometry for brain), which may partially explain the minimal overlap of candidate targets across tissues. Harmonizing platform technologies in future studies could help identify additional shared targets. Fifth, the exclusion of EHBP1 from our downstream analyses due to missing expression data may have obscured its potential relevance in PD pathophysiology. Finally, the results of prioritized protein targets were computational hypotheses based on limited direct evidence, which are relatively primary and call for experimental validation in the future. Addressing these limitations through broader proteomic profiling, larger and more diverse cohorts, and uniform assay platforms will be vital to refining our understanding of causal protein targets and their translational potential in PD.

## Author Contributions

All authors made significant contributions to this work and have approved the final manuscript. Concept and design: Chenhao Gao, Haobin Zhou, Weixuan Liang, Zhuofeng Wen, Jiewen Zhang, Chuiguo Huang, and Naijun Yuan. Data curation: Chenhao Gao, Haobin Zhou, Weixuan Liang, Zhuofeng Wen, and Chuiguo Huang. Analysis and interpretation of data: Chenhao Gao, Haobin Zhou, Weixuan Liang, Wanzhe Liao, Zhuofeng Wen, and Chuiguo Huang. Computational resources and support: Haobin Zhou, Chuiguo Huang, Jiewen Zhang, and Naijun Yuan. Writing of the original draft and reviews: Chuiguo Huang, Chenhao Gao, Wanzhe Liao, Zhixin Xie, Cailing Liao, Limin He, Jingzhang Sun, and Zhilin Chen. Editing draft and reviews: Haobin Zhou, Weixuan Liang, Zhuofeng Wen, Jiewen Zhang, Chuiguo Huang, and Naijun Yuan.

## Ethics Statement

Each cohort included in this study has been conducted using published studies and consortia providing publicly available summary statistics. All original studies have received ethical approval and agreed to participate, and summary‐level data were provided for analysis.

## Consent

The authors have nothing to report.

## Conflicts of Interest

The authors declare no conflicts of interest.

## Supporting information


**Figure S1.** Manhattan plot of brain protein pQTL and PD cognitive progression under the FUSION framework for PWAS. No significant associations were identified, and the top five proteins with the lowest *p*‐values are highlighted.
**Figure S2.** Manhattan plot of brain protein pQTL and PD motor progression under the FUSION framework for PWAS. No significant associations were identified, and the top five proteins with the lowest p‐values are highlighted.
**Figure S3.** Manhattan plot of brain protein pQTL and PD cognitive progression under the FUSION framework for PWAS. No significant associations were found, and the top five proteins with the lowest *p*‐values are highlighted.

**Table S1.** Sources of human plasma and brain pQTL data and PD‐related phenotypes GWAS summary statistics.
**Table S2.** F‐Value Statistics of SNPs in SMR Analysis.
**Table S3.** Results of the Reverse MR Steiger Test.
**Table S4.** Plasma proteins associated with PD progressions and onset identified through PWAS analysis.
**Table S5.** Plasma and brain proteins associated with PD progressions and onset identified through SMR analysis.
**Table S6.** Colocalization Results of Plasma and Brain Proteins with PD Progression and Onset.
**Table S7.** Reverse MR Results of Plasma Proteins on PD Progression and Onset.
**Table S8.** Brain proteins associated with PD progressions and onset identified through PWAS analysis.
**Table S9.** Results of proteins intervention on‐target side effects identified through PheW‐MR analysis.
**Table S10.** Whole Human Brain Gene Expression Data from 10x RNA‐seq in the ABA.
**Table S11.** Druggability Assessment of Target Proteins.

**Appendix S1.** STROBE‐MR checklist of recommended items to address in reports of Mendelian randomization studies.

## Data Availability

Publicly available datasets were analyzed in this study. This data can be found on deCODE Health study (https://www.decode.com/summarydata/), ROS/MAP study (https://www.synapse.org/#!Synapse:syn23627957), GWAS Catelog (https://www.ebi.ac.uk/gwas/home) FinnGen consortium (https://www.finngen.fi/fi), and Allen Brain Cell Atlas Public Dataset (https://allen‐brain‐cell‐atlas.s3.us‐west‐2.amazonaws.com/index.html).

## References

[1] H. Ye , L. A. Robak , M. Yu , M. Cykowski , and J. M. Shulman , “Genetics and Pathogenesis of Parkinson's Syndrome,” Annual Review of Pathology 18 (2023): 95–121, 10.1146/annurev-pathmechdis-031521-034145. PMC10290758 36100231

[2] B. R. Bloem , M. S. Okun , and C. Klein , “Parkinson's Disease,” Lancet 397, no. 10291 (2021): 2284–2303, 10.1016/S0140-6736(21)00218-X. 33848468

[3] Collaborators GBDN , “Global, Regional, and National Burden of Neurological Disorders, 1990‐2016: A Systematic Analysis for the Global Burden of Disease Study 2016,” Lancet Neurology 18, no. 5 (2019): 459–480, 10.1016/S1474-4422(18)30499-X. 30879893 PMC6459001

[4] W. Yang , J. L. Hamilton , C. Kopil , et al., “Current and Projected Future Economic Burden of Parkinson's Disease in the U.S,” NPJ Parkinson's Disease 6 (2020): 15, 10.1038/s41531-020-0117-1. PMC7347582 32665974

[5] A. H. V. Schapira , K. R. Chaudhuri , and P. Jenner , “Non‐Motor Features of Parkinson Disease,” Nature Reviews. Neuroscience 18, no. 7 (2017): 435–450, 10.1038/nrn.2017.62. 28592904

[6] C. H. Adler and T. G. Beach , “Neuropathological Basis of Nonmotor Manifestations of Parkinson's Disease,” Movement Disorders 31, no. 8 (2016): 1114–1119, 10.1002/mds.26605. 27030013 PMC4981515

[7] D. Weintraub and D. J. Burn , “Parkinson's Disease: The Quintessential Neuropsychiatric Disorder,” Movement Disorders 26, no. 6 (2011): 1022–1031, 10.1002/mds.23664. 21626547 PMC3513835

[8] H. Murakami , T. Shiraishi , T. Umehara , S. Omoto , and Y. Iguchi , “Recent Advances in Drug Therapy for Parkinson's Disease,” Internal Medicine 62, no. 1 (2023): 33–42, 10.2169/internalmedicine.8940-21. 35110492 PMC9876715

[9] N. Brandes , N. Linial , and M. Linial , “PWAS: Proteome‐Wide Association Study‐Linking Genes and Phenotypes by Functional Variation in Proteins,” Genome Biology 21, no. 1 (2020): 173, 10.1186/s13059-020-02089-x. 32665031 PMC7386203

[10] J. Zhang , D. Dutta , A. Kottgen , et al., “Plasma Proteome Analyses in Individuals of European and African Ancestry Identify Cis‐pQTLs and Models for Proteome‐Wide Association Studies,” Nature Genetics 54, no. 5 (2022): 593–602, 10.1038/s41588-022-01051-w. 35501419 PMC9236177

[11] T. S. Wingo , E. S. Gerasimov , Y. Liu , et al., “Integrating Human Brain Proteomes With Genome‐Wide Association Data Implicates Novel Proteins in Post‐Traumatic Stress Disorder,” Molecular Psychiatry 27, no. 7 (2022): 3075–3084, 10.1038/s41380-022-01544-4. 35449297 PMC9233006

[12] G. Davey Smith and G. Hemani , “Mendelian Randomization: Genetic Anchors for Causal Inference in Epidemiological Studies,” Human Molecular Genetics 23, no. R1 (2014): R89–R98, 10.1093/hmg/ddu328. 25064373 PMC4170722

[13] C. Giambartolomei , D. Vukcevic , E. E. Schadt , et al., “Bayesian Test for Colocalisation Between Pairs of Genetic Association Studies Using Summary Statistics,” PLoS Genetics 10, no. 5 (2014): e1004383, 10.1371/journal.pgen.1004383. 24830394 PMC4022491

[14] W. Zhou , J. B. Nielsen , L. G. Fritsche , et al., “Efficiently Controlling for Case‐Control Imbalance and Sample Relatedness in Large‐Scale Genetic Association Studies,” Nature Genetics 50, no. 9 (2018): 1335–1341, 10.1038/s41588-018-0184-y. 30104761 PMC6119127

[15] C. Knox , M. Wilson , C. M. Klinger , et al., “DrugBank 6.0: The DrugBank Knowledgebase for 2024,” Nucleic Acids Research 52, no. D1 (2024): D1265–D1275, 10.1093/nar/gkad976. 37953279 PMC10767804

[16] E. Ferkingstad , P. Sulem , B. A. Atlason , et al., “Large‐Scale Integration of the Plasma Proteome With Genetics and Disease,” Nature Genetics 53, no. 12 (2021): 1712–1721, 10.1038/s41588-021-00978-w. 34857953

[17] A. P. Wingo , Y. Liu , E. S. Gerasimov , et al., “Integrating Human Brain Proteomes With Genome‐Wide Association Data Implicates New Proteins in Alzheimer's Disease Pathogenesis,” Nature Genetics 53, no. 2 (2021): 143–146, 10.1038/s41588-020-00773-z. 33510477 PMC8130821

[18] M. M. X. Tan , M. A. Lawton , E. Jabbari , et al., “Genome‐Wide Association Studies of Cognitive and Motor Progression in Parkinson's Disease,” Movement Disorders 36, no. 2 (2021): 424–433, 10.1002/mds.28342. 33111402 PMC9053517

[19] M. A. Nalls , C. Blauwendraat , C. L. Vallerga , et al., “Identification of Novel Risk Loci, Causal Insights, and Heritable Risk for Parkinson's Disease: A Meta‐Analysis of Genome‐Wide Association Studies,” Lancet Neurology 18, no. 12 (2019): 1091–1102, 10.1016/S1474-4422(19)30320-5. 31701892 PMC8422160

[20] M. I. Kurki , J. Karjalainen , P. Palta , et al., “Author Correction: FinnGen Provides Genetic Insights From a Well‐Phenotyped Isolated Population,” Nature 615, no. 7952 (2023): E19, 10.1038/s41586-023-05837-8. 36829046 PMC10017492

[21] C. Zhang , F. Qin , X. Li , X. Du , and T. Li , “Identification of Novel Proteins for Lacunar Stroke by Integrating Genome‐Wide Association Data and Human Brain Proteomes,” BMC Medicine 20, no. 1 (2022): 211, 10.1186/s12916-022-02408-y. 35733147 PMC9219149

[22] A. Gusev , A. Ko , H. Shi , et al., “Integrative Approaches for Large‐Scale Transcriptome‐Wide Association Studies,” Nature Genetics 48, no. 3 (2016): 245–252, 10.1038/ng.3506. 26854917 PMC4767558

[23] Q. Dai , G. Zhou , H. Zhao , et al., “OTTERS: A Powerful TWAS Framework Leveraging Summary‐Level Reference Data,” Nature Communications 14, no. 1 (2023): 1271, 10.1038/s41467-023-36862-w. PMC9992663 36882394

[24] International Schizophrenia Consortium , S. M. Purcell , N. R. Wray , et al., “Common Polygenic Variation Contributes to Risk of Schizophrenia and Bipolar Disorder,” Nature 460, no. 7256 (2009): 748–752, 10.1038/nature08185. 19571811 PMC3912837

[25] T. S. H. Mak , R. M. Porsch , S. W. Choi , X. Zhou , and P. C. Sham , “Polygenic Scores via Penalized Regression on Summary Statistics,” Genetic Epidemiology 41, no. 6 (2017): 469–480, 10.1002/gepi.22050. 28480976

[26] P. Zeng and X. Zhou , “Non‐Parametric Genetic Prediction of Complex Traits With Latent Dirichlet Process Regression Models,” Nature Communications 8, no. 1 (2017): 456, 10.1038/s41467-017-00470-2. PMC5587666 28878256

[27] G. Zhou and H. Zhao , “A Fast and Robust Bayesian Nonparametric Method for Prediction of Complex Traits Using Summary Statistics,” PLoS Genetics 17, no. 7 (2021): e1009697, 10.1371/journal.pgen.1009697. 34310601 PMC8341714

[28] T. Ge , C. Y. Chen , Y. Ni , Y. A. Feng , and J. W. Smoller , “Polygenic Prediction via Bayesian Regression and Continuous Shrinkage Priors,” Nature Communications 10, no. 1 (2019): 1776, 10.1038/s41467-019-09718-5. PMC6467998 30992449

[29] Y. Liu , S. Chen , Z. Li , A. C. Morrison , E. Boerwinkle , and X. Lin , “ACAT: A Fast and Powerful p Value Combination Method for Rare‐Variant Analysis in Sequencing Studies,” American Journal of Human Genetics 104, no. 3 (2019): 410–421, 10.1016/j.ajhg.2019.01.002. 30849328 PMC6407498

[30] Z. Zhu , F. Zhang , H. Hu , et al., “Integration of Summary Data From GWAS and eQTL Studies Predicts Complex Trait Gene Targets,” Nature Genetics 48, no. 5 (2016): 481–487, 10.1038/ng.3538. 27019110

[31] A. Maimaiti , M. Turhon , A. Abulaiti , et al., “DNA Methylation Regulator‐Mediated Modification Patterns and Risk of Intracranial Aneurysm: A Multi‐Omics and Epigenome‐Wide Association Study Integrating Machine Learning, Mendelian Randomization, eQTL and mQTL Data,” Journal of Translational Medicine 21, no. 1 (2023): 660, 10.1186/s12967-023-04512-w. 37742034 PMC10518114

[32] A. Kurilshikov , C. Medina‐Gomez , R. Bacigalupe , et al., “Large‐Scale Association Analyses Identify Host Factors Influencing Human Gut Microbiome Composition,” Nature Genetics 53, no. 2 (2021): 156–165, 10.1038/s41588-020-00763-1. 33462485 PMC8515199

[33] G. Davey Smith , M. V. Holmes , N. M. Davies , and S. Ebrahim , “Mendel's Laws, Mendelian Randomization and Causal Inference in Observational Data: Substantive and Nomenclatural Issues,” European Journal of Epidemiology 35, no. 2 (2020): 99–111, 10.1007/s10654-020-00622-7. 32207040 PMC7125255

[34] G. D. Smith and S. Ebrahim , “‘Mendelian Randomization’: Can Genetic Epidemiology Contribute to Understanding Environmental Determinants of Disease?,” International Journal of Epidemiology 32, no. 1 (2003): 1–22, 10.1093/ije/dyg070. 12689998

[35] M. Chong , J. Sjaarda , M. Pigeyre , et al., “Novel Drug Targets for Ischemic Stroke Identified Through Mendelian Randomization Analysis of the Blood Proteome,” Circulation 140, no. 10 (2019): 819–830, 10.1161/CIRCULATIONAHA.119.040180. 31208196

[36] Y. Wang , J. Wang , Z. Yan , S. Liu , and W. Xu , “Potential Drug Targets for Asthma Identified in the Plasma and Brain Through Mendelian Randomization Analysis,” Frontiers in Immunology 14 (2023): 1240517, 10.3389/fimmu.2023.1240517. 37809092 PMC10551444

[37] K. Siletti , R. Hodge , A. Mossi Albiach , et al., “Transcriptomic Diversity of Cell Types Across the Adult Human Brain,” Science 382, no. 6667 (2023): eadd7046, 10.1126/science.add7046. 37824663

[38] C. Ruiz , M. Zitnik , and J. Leskovec , “Identification of Disease Treatment Mechanisms Through the Multiscale Interactome,” Nature Communications 12, no. 1 (2021): 1796, 10.1038/s41467-021-21770-8. PMC7979814 33741907

[39] Z. Qi , C. Zhang , H. Jian , et al., “N(1)‐Methyladenosine Modification of mRNA Regulates Neuronal Gene Expression and Oxygen Glucose Deprivation/Reoxygenation Induction,” Cell Death Discovery 9, no. 1 (2023): 159, 10.1038/s41420-023-01458-2. 37173310 PMC10182019

[40] A. Kurz , N. Rabbani , M. Walter , et al., “Alpha‐Synuclein Deficiency Leads to Increased Glyoxalase I Expression and Glycation Stress,” Cellular and Molecular Life Sciences 68, no. 4 (2011): 721–733, 10.1007/s00018-010-0483-7. 20711648 PMC3029823

[41] S. Sjodin , G. Brinkmalm , A. Ohrfelt , et al., “Endo‐Lysosomal Proteins and Ubiquitin CSF Concentrations in Alzheimer's and Parkinson's Disease,” Alzheimer's Research & Therapy 11, no. 1 (2019): 82, 10.1186/s13195-019-0533-9. PMC6745076 31521194

[42] C. M. Qiao , X. Y. Ma , L. L. Tan , et al., “Indoleamine 2, 3‐Dioxygenase 1 Inhibition Mediates the Therapeutic Effects in Parkinson's Disease Mice by Modulating Inflammation and Neurogenesis in a Gut Microbiota Dependent Manner,” Experimental Neurology 385 (2025): 115142, 10.1016/j.expneurol.2025.115142. 39793693

[43] Q. Sun , Y. J. Li , and S. B. Ning , “Investigating the Molecular Mechanisms Underlying the Co‐Occurrence of Parkinson's Disease and Inflammatory Bowel Disease Through the Integration of Multiple Datasets,” Scientific Reports 14, no. 1 (2024): 17028, 10.1038/s41598-024-67890-1. 39043798 PMC11266657

[44] A. Venkatraman , E. Murugan , S. J. Lin , G. S. L. Peh , L. Rajamani , and J. S. Mehta , “Effect of Osmolytes on In‐Vitro Aggregation Properties of Peptides Derived From TGFBIp,” Scientific Reports 10, no. 1 (2020): 4011, 10.1038/s41598-020-60944-0. 32132634 PMC7055237

[45] J. Liu , Y. Wang , Y. Zhao , et al., “Comprehensive Variant Analysis of Phospholipase A2 Superfamily Genes in Large Chinese Parkinson' s Disease Cohorts,” Mechanisms of Ageing and Development 219 (2024): 111940, 10.1016/j.mad.2024.111940. 38750970

[46] K. Senkevich , M. Beletskaia , A. Dworkind , et al., “Association of Rare Variants in ARSA With Parkinson's Disease,” Movement Disorders 38, no. 10 (2023): 1806–1812, 10.1002/mds.29521. 37381728 PMC10615669

[47] X. J. Gu , W. M. Su , M. Dou , et al., “Expanding Causal Genes for Parkinson's Disease via Multi‐Omics Analysis,” NPJ Parkinson's Disease 9, no. 1 (2023): 146, 10.1038/s41531-023-00591-0. PMC10590374 37865667

[48] D. Wu , X. Bi , and K. H. Chow , “Identification of Female‐Enriched and Disease‐Associated Microglia (FDAMic) Contributes to Sexual Dimorphism in Late‐Onset Alzheimer's Disease,” Journal of Neuroinflammation 21, no. 1 (2024): 1, 10.1186/s12974-023-02987-4. 38178204 PMC10765928

[49] D. M. Sherry , A. R. Murray , Y. Kanan , et al., “Lack of Protein‐Tyrosine Sulfation Disrupts Photoreceptor Outer Segment Morphogenesis, Retinal Function and Retinal Anatomy,” European Journal of Neuroscience 32, no. 9 (2010): 1461–1472, 10.1111/j.1460-9568.2010.07431.x. 21039965 PMC3058723

[50] O. Kwon , D. Kwak , S. H. Ha , et al., “Nudix‐Type Motif 2 Contributes to Cancer Proliferation Through the Regulation of Rag GTPase‐Mediated Mammalian Target of Rapamycin Complex 1 Localization,” Cellular Signalling 32 (2017): 24–35, 10.1016/j.cellsig.2017.01.015. 28089905

[51] D. Li , Q. Xie , J. Xie , et al., “Cerebrospinal Fluid Proteomics Identifies Potential Biomarkers for Early‐Onset Alzheimer's Disease,” Journal of Alzheimer's Disease 100, no. 1 (2024): 261–277, 10.3233/JAD-240022. 38848183

[52] A. E. Visser , N. M. de Vries , E. Richard , and B. R. Bloem , “Tackling Vascular Risk Factors as a Possible Disease Modifying Intervention in Parkinson's Disease,” NPJ Parkinson's Disease 10, no. 1 (2024): 50, 10.1038/s41531-024-00666-6. PMC10908840 38431725

[53] A. Salminen , “Role of Indoleamine 2,3‐Dioxygenase 1 (IDO1) and Kynurenine Pathway in the Regulation of the Aging Process,” Ageing Research Reviews 75 (2022): 101573, 10.1016/j.arr.2022.101573. 35085834

[54] B. Hervier , M. Ribon , N. Tarantino , et al., “Increased Concentrations of Circulating Soluble MHC Class I‐Related Chain A (sMICA) and sMICB and Modulation of Plasma Membrane MICA Expression: Potential Mechanisms and Correlation With Natural Killer Cell Activity in Systemic Lupus Erythematosus,” Frontiers in Immunology 12 (2021): 633658, 10.3389/fimmu.2021.633658. 34012432 PMC8126610

[55] Y. Ran , K. Wu , C. Hu , et al., “Downregulated APOD and FCGR2A Correlates With Immune Infiltration and Lipid‐Induced Symptoms of Irritable Bowel Syndrome,” Scientific Reports 13, no. 1 (2023): 14211, 10.1038/s41598-023-41004-9. 37648784 PMC10469184

[56] H. Li , D. Yang , M. Hao , and H. Liu , “Differential Expression of HAVCR2 Gene in Pan‐Cancer: A Potential Biomarker for Survival and Immunotherapy,” Frontiers in Genetics 13 (2022): 972664, 10.3389/fgene.2022.972664. 36081997 PMC9445440

[57] D. A. Braun , K. Street , K. P. Burke , et al., “Progressive Immune Dysfunction With Advancing Disease Stage in Renal Cell Carcinoma,” Cancer Cell 39, no. 5 (2021): 632–648 e8, 10.1016/j.ccell.2021.02.013. 33711273 PMC8138872

