# Supplementary material for: Proteome‐Wide Association Study for Finding Druggable Targets in Progression and Onset of Parkinson's Disease
Source: CNS Neurosci Ther. 2025 Feb 26;31(2):e70294. doi: 10.1111/cns.70294 (PMC11862824; doi:10.1111/cns.70294)
Supplement: Supplementary file 1 — Figure S1. Manhattan plot of brain protein pQTL and PD cognitive progression under the FUSION framework for PWAS. No significant associations were identified, and the top five proteins with the lowest p‐values are highlighted. Figure S2. Manhattan plot of brain protein pQTL and PD motor progression under the FUSION framework for PWAS. No significant associations were identified, and the top five proteins with the lowest p‐values are highlighted. Figure S3. Manhattan plot of brain protein pQTL and PD cognitive progression under the FUSION framework for PWAS. No significant associations were found, and the top five proteins with the lowest p‐values are highlighted. [file CNS-31-e70294-s001.docx]

**Supplementary Figure**

**Figure S1.** Manhattan plot of brain protein pQTL and PD cognitive progression under the FUSION framework for PWAS. No significant associations were identified, and the top five proteins with the lowest p-values are highlighted.

**Figure S2.** Manhattan plot of brain protein pQTL and PD motor progression under the FUSION framework for PWAS. No significant associations were identified, and the top five proteins with the lowest p-values are highlighted.

**Figure S3.** Manhattan plot of brain protein pQTL and PD cognitive progression under the FUSION framework for PWAS. No significant associations were found, and the top five proteins with the lowest p-values are highlighted.
