# Supplementary material for: Proteome‐Wide Association Study for Finding Druggable Targets in Progression and Onset of Parkinson's Disease
Source: CNS Neurosci Ther. 2025 Feb 26;31(2):e70294. doi: 10.1111/cns.70294 (PMC11862824; doi:10.1111/cns.70294)
Supplement: Supplementary file 3 — Appendix S1. STROBE‐MR checklist of recommended items to address in reports of Mendelian randomization studies. [file CNS-31-e70294-s003.docx]

**STROBE-MR checklist of recommended items to address in reports of Mendelian randomization studies**^1^ ^2^

| **Item No.** | **Section** | **Checklist item** | **Page No.** | **Relevant text from manuscript** |
| --- | --- | --- | --- | --- |
| 1 | **TITLE and ABSTRACT** | Indicate Mendelian randomization (MR) as the study’s design in the title and/or the abstract if that is a main purpose of the study | 2 | Significant protein associations (FDR-corrected p < 0.05) from PWAS were further validated using summary-based Mendelian randomization (SMR), colocalization analyses, and reverse Mendelian randomization (MR) to establish causality. Phenome-wide Mendelian randomization (PheW-MR) was performed to assess potential side effects across 679 disease traits when targeting these proteins to reduce PD-related phenotype risk by 20%. |
|  | **INTRODUCTION** |  |  |  |
| 2 | **Background** | Explain the scientific background and rationale for the reported study. What is the exposure? Is a potential causal relationship between exposure and outcome plausible? Justify why MR is a helpful method to address the study question | 2, 3, 4 | Parkinson's disease (PD) is a neurodegenerative disorder characterized by the progressive loss of dopaminergic neurons in the substantia nigra pars compacta and the accumulation of α-synuclein aggregates, known as Lewy bodies. It is the second most prevalent neurodegenerative disease after Alzheimer's disease. Epidemiological studies indicate a global increase in PD cases, rising from 2.5 million to 6.1 million over the past three decades. With the aging global population, the incidence of PD is projected to escalate significantly, imposing substantial socio-economic burdens on patients, and healthcare systems.  PD manifests through a spectrum of motor symptoms, including tremors, rigidity, bradykinesia, and postural instability, resulting from the degeneration of dopaminergic neurons. In addition to these motor deficits, PD encompasses a range of non-motor symptoms such as cognitive decline, mood disorders, and autonomic dysfunction, which contribute to the disease's complexity and severely impact the quality of life of affected individuals. The heterogeneity in disease progression, characterized by varying rates of motor and cognitive deterioration among patients, presents significant challenges for effective treatment and management strategies. Current therapeutic approaches for PD primarily aim at symptomatic relief, employing medications like levodopa and dopamine agonists to replenish dopamine levels and alleviate motor symptoms. While these treatments can provide temporary improvement, they do not halt the underlying neurodegenerative processes driving the disease. The absence of disease-modifying therapies underscores the urgent need for interventions that can influence both the onset and progression of PD.  Specifically, plasma and brain proteomics offer valuable insights into systemic and central nervous system-specific protein alterations linked to PD. Integrative genomic analyses that combine genome-wide association studies (GWAS) with proteomic data enable the elucidation of causal relationships between genetic variants, protein expression, and disease traits. Methodologies such as PWAS, summary-based Mendelian randomization (SMR), colocalization analyses, and phenome-wide Mendelian randomization (PheW-MR) |
| 3 | **Objectives** | State specific objectives clearly, including pre-specified causal hypotheses (if any). State that MR is a method that, under specific assumptions, intends to estimate causal effects | 2,3 | Advancements in proteomics and genomics have opened new avenues for identifying biomarkers and therapeutic targets in complex diseases such as PD. Proteome-wide association studies (PWAS), leveraging protein quantitative trait loci (pQTL) data, facilitate the identification of protein-level associations with disease phenotypes9. Specifically, plasma and brain proteomics offer valuable insights into systemic and central nervous system-specific protein alterations linked to PD. Integrative genomic analyses that combine genome-wide association studies (GWAS) with proteomic data enable the elucidation of causal relationships between genetic variants, protein expression, and disease traits. Methodologies such as PWAS, summary-based Mendelian randomization (SMR), colocalization analyses, and phenome-wide Mendelian randomization (PheW-MR) are instrumental in dissecting the genetic architecture of PD and identifying proteins that may serve as potential therapeutic targets. Therefore, by integrating these powerful and steady approaches in a logical order, our study aims to identify latent but reliable drug targets for PD. |
|  | **METHODS** |  |  |  |
| 4 | **Study design and data sources** | Present key elements of the study design early in the article. Consider including a table listing sources of data for all phases of the study. For each data source contributing to the analysis, describe the following: |  |  |
|  | a) | Setting: Describe the study design and the underlying population, if possible. Describe the setting, locations, and relevant dates, including periods of recruitment, exposure, follow-up, and data collection, when available. | 5, 6 | 1. Data Sources  We obtained plasma pQTL data from the deCODE Health study, which performed comprehensive proteomic profiling in plasma samples from 35,559 Icelandic participants using the SomaScan platform, ultimately quantifying 4,907 distinct plasma proteins. For brain-derived protein data, we used pQTL information on 1,097 proteins measured in the dorsolateral prefrontal cortex from participants in the ROS/MAP using mass spectrometry. We also incorporated GWAS summary statistics for three PD progression phenotypes, including composite (2,755 patients), motor (2,848 patients), and cognitive (2,788 patients), as reported by Tan MMX et al. For PD onset, the discovery cohort consisted of GWAS summary statistics derived from Nalls MA et al.18 (15,056 cases and 12,637 controls), and the replication cohort employed data from the FinnGen consortium19 (4,235 cases and 373,042 controls). Details of these datasets are provided in Supplementary Table S1. |
|  | b) | Participants: Give the eligibility criteria, and the sources and methods of selection of participants. Report the sample size, and whether any power or sample size calculations were carried out prior to the main analysis | 5,6 | 1. Data Sources  We obtained plasma pQTL data from the deCODE Health study, which performed comprehensive proteomic profiling in plasma samples from 35,559 Icelandic participants using the SomaScan platform, ultimately quantifying 4,907 distinct plasma proteins. For brain-derived protein data, we used pQTL information on 1,097 proteins measured in the dorsolateral prefrontal cortex from participants in the ROS/MAP using mass spectrometry. We also incorporated GWAS summary statistics for three PD progression phenotypes, including composite (2,755 patients), motor (2,848 patients), and cognitive (2,788 patients), as reported by Tan MMX et al. For PD onset, the discovery cohort consisted of GWAS summary statistics derived from Nalls MA et al.18 (15,056 cases and 12,637 controls), and the replication cohort employed data from the FinnGen consortium19 (4,235 cases and 373,042 controls). Details of these datasets are provided in Supplementary Table S1. |
|  | c) | Describe measurement, quality control and selection of genetic variants | 7, 9,10 | **Summary-based Mendelian randomization (SMR) analysis**  To rigorously validate our PWAS findings, we employed SMR to confirm both brain and plasma proteins found to be causally associated with PD-related phenotypes. SMR integrates pQTL and GWAS summary statistics within the MR framework, which utilizes instrumental variables (IVs), genetic variants that serve as proxies for protein levels, to enable the assessment of the causal impact of protein levels on PD-related traits. Unlike conventional two-sample MR, where two independent GWAS datasets are required to estimate the causal effect between traits, SMR combines pQTL and GWAS data and utilizes the Heterogeneity in Dependent Instruments (HEIDI) test. This approach offers more robust discrimination between pleiotropic and linkage effects, reduces potential biases due to LD, and lowers the large sample size requirements often seen in standard MR methods.  Reverse MR analysis  To ensure an adequate number of instrumental variables (IVs) for each PD-related phenotype, we adopted a relaxed significance threshold of P < 5e-06 and performed LD clumping to maintain LD independence (r² < 0.001, window size = 10,000 kb) among the SNPs.  Subsequently, we calculated F-statistics for each IV to assess their strength, excluding those with F-values < 10 to mitigate weak instrument bias.  Phenome-Wide Mendelian Randomization (PheW-MR) Analysis of 679 Disease Traits  To determine the effect sizes of proteins on the 679 diseases, we performed MR. In this process, IVs were selected using a stringent significance threshold of P < 5e-08, followed by LD clumping (r² < 0.1, window size = 10,000 kb) to ensure LD independence among the selected SNPs. |
|  | d) | For each exposure, outcome, and other relevant variables, describe methods of assessment and diagnostic criteria for diseases | / | / |
|  | e) | Provide details of ethics committee approval and participant informed consent, if relevant | / | / |
| 5 | **Assumptions** | Explicitly state the three core IV assumptions for the main analysis (relevance, independence and exclusion restriction) as well assumptions for any additional or sensitivity analysis | 7 | SMR is an extension of MR, and MR adheres to three core assumptions: (i) the relevance assumption, which requires a strong association between IVs and the exposure; (ii) the independence assumption, stating that IVs influence the outcome solely through the exposure; (iii) the exclusion restriction assumption, which dictates that IVs should not have a direct impact on the outcome |
| 6 | **Statistical methods: main analysis** | Describe statistical methods and statistics used |  |  |
|  | a) | Describe how quantitative variables were handled in the analyses (i.e., scale, units, model) | 7,9,10 | Summary-based Mendelian randomization (SMR) analysis  The threshold for the P-value of the IVs was 5e-08 when running SMR. To ensure the robustness of our instrumental variables (IVs), we calculated F-statistics using the established formula (10.1038/s41588-020-00763-1):  F=$\frac{r^{2}\left( N-2 \right)}{\left( 1-r^{2} \right)}$  where N is the sample size and r2 is the proportion of variance in the exposure explained by the IV. An F-statistic greater than 10 is commonly regarded as indicative of sufficient IV strength, thus mitigating weak instrument bias. All calculated F-values are presented in Supplementary Tables S2.  Reverse MR analysis  To ensure an adequate number of instrumental variables (IVs) for each PD-related phenotype, we adopted a relaxed significance threshold of *P* < 5e-06 and performed LD clumping to maintain LD independence (r² < 0.001, window size = 10,000 kb) among the SNPs.  **Phenome-Wide Mendelian Randomization**  To determine the effect sizes of proteins on the 679 diseases, we performed MR. In this process, IVs were selected using a stringent significance threshold of *P* < 5e-08, followed by LD clumping (r² < 0.1, window size = 10,000 kb) to ensure LD independence among the selected SNPs. |
|  | b) | Describe how genetic variants were handled in the analyses and, if applicable, how their weights were selected | 7,8,9,10 | Summary-based Mendelian randomization (SMR) analysis  The threshold for the P-value of the IVs was 5e-08 when running SMR. To ensure the robustness of our instrumental variables (IVs), we calculated F-statistics using the established formula (10.1038/s41588-020-00763-1):  F=$\frac{r^{2}\left( N-2 \right)}{\left( 1-r^{2} \right)}$  where N is the sample size and r2 is the proportion of variance in the exposure explained by the IV. An F-statistic greater than 10 is commonly regarded as indicative of sufficient IV strength, thus mitigating weak instrument bias. All calculated F-values are presented in Supplementary Tables S2.  Reverse MR analysis  To ensure an adequate number of instrumental variables (IVs) for each PD-related phenotype, we adopted a relaxed significance threshold of *P* < 5e-06 and performed LD clumping to maintain LD independence (r² < 0.001, window size = 10,000 kb) among the SNPs.  **Phenome-Wide Mendelian Randomization (PheW-MR) Analysis of 679 Disease Traits**  To determine the effect sizes of proteins on the 679 diseases, we performed MR. In this process, IVs were selected using a stringent significance threshold of *P* < 5e-08, followed by LD clumping (r² < 0.1, window size = 10,000 kb) to ensure LD independence among the selected SNPs. |
|  | c) | Describe the MR estimator (e.g. two-stage least squares, Wald ratio) and related statistics. Detail the included covariates and, in case of two-sample MR, whether the same covariate set was used for adjustment in the two samples | 7,8,9,10 | **Summary-based Mendelian randomization (SMR) analysis**  We adopted the SMR-derived estimates as our primary measures of each protein’s influence on PD-related phenotypes. Given the inherent stringency of the SMR method, we applied the Benjamini-Hochberg procedure to control the false discovery rate (FDR), thereby minimizing false positives without excessively inflating false negatives. Any protein that met the criteria of an FDR-adjusted SMR *P*-values < 0.05 and a HEIDI *P*-values > 0.01 was considered to have a causal relationship with the respective PD-related phenotype.  Reverse MR analysis  Subsequently, we calculated F-statistics for each IV to assess their strength, excluding those with F-values < 10 to mitigate weak instrument bias. Following this, the Steiger test was conducted to verify the directionality of the associations, retaining only SNPs that explained a larger proportion of variance in the exposure compared to the outcome. This step ensured that each IV primarily influenced the outcome through its effect on the exposure. Finally, we applied a Bonferroni-corrected significance threshold of *P*< 0.05 for the reverse MR analysis. Associations surpassing this threshold were considered statistically significant, thereby identifying potential bidirectional causal links between PD-related phenotypes and the candidate proteins.  **Phenome-Wide Mendelian Randomization (PheW-MR) Analysis of 679 Disease Traits**  The effects of proteins on PD-related phenotypes were obtained from SMR analyses linking the candidate proteins to PD-related traits. We defined a side effect as any influence on an alternate disease trait resulting from manipulating a target protein to achieve a 20% reduction in the risk of the PD-related phenotype. To estimate and standardize the magnitude of side effects, we adopted the following formula for the odds ratio of the effect (OR_effect):  $\mathrm{OR}_{\mathrm{effect}}=exp \left( \beta_{\mathrm{effect}} \right)$  where  $\beta_{effect}=\frac{\beta_{679 diseases}}{\beta_{PD phenotypes}}\times ln \left( 0.8 \right)$  Here, $\beta_{679 diseases}$ represents the effect of the candidate proteins on the 679 diseases, with only associations having *P*-values below 0.05 included. $\beta_{PD phenotypes}$denotes the proteins’ effect on PD-related phenotypes, derived from the PheW-MR and SMR analyses. Proteins with OR values greater than 1 were considered to have potentially adverse side effects, whereas those with OR values less than 1 were deemed to confer beneficial side effects. P-values were estimated using a bootstrap method with one million iterations (n = 1,000,000), and associations achieving Bonferroni-corrected *P*-values < 0.05 were regarded as statistically significant. |
|  | d) | Explain how missing data were addressed | / | / |
|  | e) | If applicable, indicate how multiple testing was addressed |  |  |
| 7 | **Assessment of assumptions** | Describe any methods or prior knowledge used to assess the assumptions or justify their validity | 7 | SMR is an extension of MR, and MR adheres to three core assumptions: (i) the relevance assumption, which requires a strong association between IVs and the exposure; (ii) the independence assumption, stating that IVs influence the outcome solely through the exposure; (iii) the exclusion restriction assumption, which dictates that IVs should not have a direct impact on the outcome |
| 8 | **Sensitivity analyses and additional analyses** | Describe any sensitivity analyses or additional analyses performed (e.g. comparison of effect estimates from different approaches, independent replication, bias analytic techniques, validation of instruments, simulations) | 7,8,9,10 | **Summary-based Mendelian randomization (SMR) analysis**  We adopted the SMR-derived estimates as our primary measures of each protein’s influence on PD-related phenotypes. Given the inherent stringency of the SMR method, we applied the Benjamini-Hochberg procedure to control the false discovery rate (FDR), thereby minimizing false positives without excessively inflating false negatives. Any protein that met the criteria of an FDR-adjusted SMR *P*-values < 0.05 and a HEIDI *P*-values > 0.01 was considered to have a causal relationship with the respective PD-related phenotype.  Reverse MR analysis  Subsequently, we calculated F-statistics for each IV to assess their strength, excluding those with F-values < 10 to mitigate weak instrument bias. Following this, the Steiger test was conducted to verify the directionality of the associations, retaining only SNPs that explained a larger proportion of variance in the exposure compared to the outcome. This step ensured that each IV primarily influenced the outcome through its effect on the exposure. Finally, we applied a Bonferroni-corrected significance threshold of *P*< 0.05 for the reverse MR analysis. Associations surpassing this threshold were considered statistically significant, thereby identifying potential bidirectional causal links between PD-related phenotypes and the candidate proteins.  **Phenome-Wide Mendelian Randomization (PheW-MR) Analysis of 679 Disease Traits**  The effects of proteins on PD-related phenotypes were obtained from SMR analyses linking the candidate proteins to PD-related traits. We defined a side effect as any influence on an alternate disease trait resulting from manipulating a target protein to achieve a 20% reduction in the risk of the PD-related phenotype. To estimate and standardize the magnitude of side effects, we adopted the following formula for the odds ratio of the effect (OR_effect):  $\mathrm{OR}_{\mathrm{effect}}=exp \left( \beta_{\mathrm{effect}} \right)$  where  $\beta_{effect}=\frac{\beta_{679 diseases}}{\beta_{PD phenotypes}}\times ln \left( 0.8 \right)$  Here, $\beta_{679 diseases}$ represents the effect of the candidate proteins on the 679 diseases, with only associations having *P*-values below 0.05 included. $\beta_{PD phenotypes}$denotes the proteins’ effect on PD-related phenotypes, derived from the PheW-MR and SMR analyses. Proteins with OR values greater than 1 were considered to have potentially adverse side effects, whereas those with OR values less than 1 were deemed to confer beneficial side effects. P-values were estimated using a bootstrap method with one million iterations (n = 1,000,000), and associations achieving Bonferroni-corrected *P*-values < 0.05 were regarded as statistically significant. |
| 9 | **Software and pre-registration** |  |  |  |
|  | a) | Name statistical software and package(s), including version and settings used | / | / |
|  | b) | State whether the study protocol and details were pre-registered (as well as when and where) | 30 | Each cohort included in this study has been conducted using published studies and consortia providing publicly available summary statistics. All original studies have received ethical approval and agreed to participate, and summary-level data were provided for analysis. |
|  | **RESULTS** |  |  |  |
| 10 | **Descriptive data** |  |  |  |
|  | a) | Report the numbers of individuals at each stage of included studies and reasons for exclusion. Consider use of a flow diagram | 5, 6 | **1. Data Sources**  We obtained plasma pQTL data from the deCODE Health study, which performed comprehensive proteomic profiling in plasma samples from 35,559 Icelandic participants using the SomaScan platform, ultimately quantifying 4,907 distinct plasma proteins^16^. For brain-derived protein data, we used pQTL information on 1,097 proteins measured in the dorsolateral prefrontal cortex from participants in the ROS/MAP using mass spectrometry^17^. We also incorporated GWAS summary statistics for three PD progression phenotypes, including composite (2,755 patients), motor (2,848 patients), and cognitive (2,788 patients), as reported by Tan MMX et al.^18^ For PD onset, the discovery cohort consisted of GWAS summary statistics derived from Nalls MA et al.^19^ (15,056 cases and 12,637 controls), and the replication cohort employed data from the FinnGen consortium^20^ (4,235 cases and 373,042 controls). Details of these datasets are provided in **Supplementary Table S1**. |
|  | b) | Report summary statistics for phenotypic exposure(s), outcome(s), and other relevant variables (e.g. means, SDs, proportions) | 5,6 | **1. Data Sources**  We obtained plasma pQTL data from the deCODE Health study, which performed comprehensive proteomic profiling in plasma samples from 35,559 Icelandic participants using the SomaScan platform, ultimately quantifying 4,907 distinct plasma proteins^16^. For brain-derived protein data, we used pQTL information on 1,097 proteins measured in the dorsolateral prefrontal cortex from participants in the ROS/MAP using mass spectrometry^17^. We also incorporated GWAS summary statistics for three PD progression phenotypes, including composite (2,755 patients), motor (2,848 patients), and cognitive (2,788 patients), as reported by Tan MMX et al.^18^ For PD onset, the discovery cohort consisted of GWAS summary statistics derived from Nalls MA et al.^19^ (15,056 cases and 12,637 controls), and the replication cohort employed data from the FinnGen consortium^20^ (4,235 cases and 373,042 controls). Details of these datasets are provided in **Supplementary Table S1**. |
|  | c) | If the data sources include meta-analyses of previous studies, provide the assessments of heterogeneity across these studies | 5,6 | **1. Data Sources**  We obtained plasma pQTL data from the deCODE Health study, which performed comprehensive proteomic profiling in plasma samples from 35,559 Icelandic participants using the SomaScan platform, ultimately quantifying 4,907 distinct plasma proteins^16^. For brain-derived protein data, we used pQTL information on 1,097 proteins measured in the dorsolateral prefrontal cortex from participants in the ROS/MAP using mass spectrometry^17^. We also incorporated GWAS summary statistics for three PD progression phenotypes, including composite (2,755 patients), motor (2,848 patients), and cognitive (2,788 patients), as reported by Tan MMX et al.^18^ For PD onset, the discovery cohort consisted of GWAS summary statistics derived from Nalls MA et al.^19^ (15,056 cases and 12,637 controls), and the replication cohort employed data from the FinnGen consortium^20^ (4,235 cases and 373,042 controls). Details of these datasets are provided in **Supplementary Table S1**. |
|  | d) | For two-sample MR:  i.  Provide justification of the similarity of the genetic variant-exposure associations between the exposure and outcome samples  ii.  Provide information on the number of individuals who overlap between the exposure and outcome studies | 5,6 | **1. Data Sources**  We obtained plasma pQTL data from the deCODE Health study, which performed comprehensive proteomic profiling in plasma samples from 35,559 Icelandic participants using the SomaScan platform, ultimately quantifying 4,907 distinct plasma proteins^16^. For brain-derived protein data, we used pQTL information on 1,097 proteins measured in the dorsolateral prefrontal cortex from participants in the ROS/MAP using mass spectrometry^17^. We also incorporated GWAS summary statistics for three PD progression phenotypes, including composite (2,755 patients), motor (2,848 patients), and cognitive (2,788 patients), as reported by Tan MMX et al.^18^ For PD onset, the discovery cohort consisted of GWAS summary statistics derived from Nalls MA et al.^19^ (15,056 cases and 12,637 controls), and the replication cohort employed data from the FinnGen consortium^20^ (4,235 cases and 373,042 controls). Details of these datasets are provided in **Supplementary Table S1**. |
| 11 | **Main results** |  |  |  |
|  | a) | Report the associations between genetic variant and exposure, and between genetic variant and outcome, preferably on an interpretable scale | 7,8,9,10 | SMR  The threshold for the P-value of the IVs was 5e-08 when running SMR. To ensure the robustness of our instrumental variables (IVs), we calculated F-statistics using the established formula^31^:  F=$\frac{r^{2}\left( N-2 \right)}{\left( 1-r^{2} \right)}$  where N is the sample size and r2 is the proportion of variance in the exposure explained by the IV. An F-statistic greater than 10 is commonly regarded as indicative of sufficient IV strength, thus mitigating weak instrument bias. All calculated F-values are presented in **Supplementary Tables S2**.  Reverse MR analysis  To ensure an adequate number of instrumental variables (IVs) for each PD-related phenotype, we adopted a relaxed significance threshold of *P* < 5e-06 and performed LD clumping to maintain LD independence (r² < 0.001, window size = 10,000 kb) among the SNPs.  Subsequently, we calculated F-statistics for each IV to assess their strength, excluding those with F-values < 10 to mitigate weak instrument bias. Following this, the Steiger test was conducted to verify the directionality of the associations, retaining only SNPs that explained a larger proportion of variance in the exposure compared to the outcome  **PheW-MR**  To determine the effect sizes of proteins on the 679 diseases, we performed MR. In this process, IVs were selected using a stringent significance threshold of *P* < 5e-08, followed by LD clumping (r² < 0.1, window size = 10,000 kb) to ensure LD independence among the selected SNPs. |
|  | b) | Report MR estimates of the relationship between exposure and outcome, and the measures of uncertainty from the MR analysis, on an interpretable scale, such as odds ratio or relative risk per SD difference | 14,15,16,17,22,23 | To validate the causal associations between plasma proteins and the PD progression phenotypes, we performed SMR and HEIDI analyses. Applying stringent criteria—SMR P-value (FDR-corrected) < 0.05 and HEIDI P-value > 0.05—we identified 12 plasma proteins causally associated with cognitive progression, 5 with motor progression, and 6 with composite progression (Supplementary Table S5, Figure 3). A subsequent colocalization analysis (PPH4 > 0.8) confirmed that, of the 12 proteins linked to cognitive progression, 10 (ALKBH3, GLO1, IDO1, SERPINA3, SORD, TPST1, GM2A, MICB, SH3BGRL3, and TGFBI) shared causal variants with PD cognitive progression loci (Supplementary Table S6, Figure 3). Among these 10 proteins, the abundance of ALKBH3 (β = 0.482, P=2.58e-03), GLO1 (β = 1.094, P=3.64e-03), IDO1 (β = 1.899, P=7.34e-03), SERPINA3 (β = 0.381, P=9.87e-03), SORD (β = 0.521, P=2.17e-02), and TPST1 (β = 0.342, P=1.10e-02) exhibited significant positive causal correlations with cognitive progression, whereas GM2A (β = -0.414, P=6.48e-03), MICB (β = -0.105, P=4.79e-02), SH3BGRL3 (β = -0.402, P=1.36e-02), and TGFBI (β = -0.247, P=1.76e-03) demonstrated negative correlations. Of the 5 proteins associated with motor progression, 4 (NUDT2, PLA2G12B, EVA1C, and MATN3) passed the colocalization test. Specifically, the abundance of NUDT2 (β = 0.378, P=1.11e-02) and PLA2G12B (β = 0.968, P=2.90e-02) correlated positively with motor progression, whereas EVA1C (β = -1.446, P=9.73e-03) and MATN3 (β = -0.160, P=1.69e-02) correlated negatively. Additionally, among the 6 proteins implicated in composite progression, 3 (SH3BGRL3, NANS, and RSPO3) were validated by colocalization analysis. SH3BGRL3 (β = 0.392, P=3.84e-02) was positively correlated with composite progression, while NANS (β = -1.153, P=1.78e-02) and RSPO3 (β = -0.503, P=6.19e-03) exhibited negative correlations. Notably, SH3BGRL3 emerged as a causal factor in both cognitive and composite progressions, despite manifesting a negative correlation with the former and a positive correlation with the latter. Finally, reverse MR analyses revealed no significant bidirectional associations (P < 0.05 / 114) between these proteins and the three PD progression phenotypes (Supplementary Table S7).  We applied SMR and the HEIDI test to these 54 proteins to explore their causal associations with PD onset. Overall, 22 plasma proteins demonstrated significant causal evidence for PD onset (*P_SMR_* *_(FDR-corrected_*_)_< 0.05 and *P_HEIDI_* > 0.01) (**Supplementary Table S5**). Colocalization analysis confirmed that 9 of these proteins (ARSA, EHBP1, FCGR2A, GGH, GPNMB, HDHD2, DNAJB4, HAVCR2, and PDCD1LG2) shared a common causal variant with PD onset (PP_H4_ > 0.8; **Supplementary Table S6**, Figure 4B). Among these 9 proteins, the abundance of 6 proteins, ARSA (OR = 2.177*,P*=7.53e-04), EHBP1 (OR = 3.739, *P*=1.27e-02), FCGR2A (OR = 1.059, *P*=4.37e-05), GGH (OR = 1.167, *P*=2.81e-02), GPNMB (OR = 1.503, *P*=1.79e-07), and HDHD2 (OR = 1.230, *P*=1.64e-02), was significantly associated with an elevated risk of PD onset, whereas the abundance of DNAJB4 (OR = 0.701*, P*=2.54e-03), HAVCR2 (OR = 0.905, *P*=5.55e-03), and PDCD1LG2 (OR = 0.852, *P*=2.44e-02) was associated with a reduced risk. To examine potential bidirectional causal relationships, we also performed reverse MR analyses but detected no significant associations, reinforcing the robustness of the observed causal links between the identified proteins and PD onset (**Supplementary Table S7**).  For the four proteins validated through PWAS, we initially performed SMR and the HEIDI test to elucidate their causal relationships with PD onset (**Supplementary Table S5**). Among these four proteins, only GPNMB and CD38 had valid IVs extracted from brain pQTL data. Consequently, we conducted SMR and HEIDI tests exclusively for these two proteins. The results revealed that both GPNMB and CD38 exhibited significant causal associations with PD onset. Specifically, the abundance of GPNMB (OR = 1.394, *P*=7.73e-07) was associated with an increased risk of PD onset, whereas the abundance of CD38 (OR = 0.415, *P*=3.32e-08) was associated with a decreased risk. Subsequent colocalization analysis using the COLOC method confirmed the associations between these two proteins and PD onset (**Supplementary Table S6**). The analysis showed that only GPNMB had PP.H4 exceeding 0.8, indicating a shared causal variant between GPNMB and PD onset. As a final sensitivity analysis, we attempted to perform a reverse MR analysis to investigate the association between GPNMB and PD onset. Unfortunately, because we only had SNPs within the GPNMB cis region, and there was no overlap with the IVs for PD onset, we did not have valid IVs for the analysis, thereby precluding the reverse MR analysis for this protein. This limitation prevents us from fully establishing the bidirectional causal relationship of this protein, necessitating further investigation in future studies.  Following the identification of candidate targets associated with PD-related phenotypes, we conducted a comprehensive analysis across 679 common disease traits to characterize the side-effect profiles of each prioritized protein as a potential therapeutic target. Unlike the previous SMR approach, PheW-MR results were standardized to reflect a 20% reduction in the risk of PD-related phenotypes mediated by each protein. Consequently, the observed associations can be interpreted as potential side effects that may arise from therapeutically targeting these proteins.  Under this 20% risk-reduction assumption, PheW-MR analyses (*P*< 0.05 / 3126) identified 1,529 significant beneficial side effects (83.7%) and 297 adverse side effects (16.3%) across 25 candidate targets. A paired t-test confirmed that beneficial side effects significantly outnumbered adverse side effects (*P* = 7.91e-05), suggesting that the majority of identified side effects were beneficial (**Supplementary Table S9, Figure 7**). Of these 25 candidate targets, 17 exhibited exclusively beneficial side effects, while the remaining 8 displayed both beneficial and adverse side effects.  Among the 17 candidate targets with exclusively beneficial side effects, we focused on those that reduce the risk of four major PD progression phenotypes and demonstrated the largest number of positive outcomes. For targets mitigating PD cognitive progression, MICB exhibited the most pronounced beneficial profile, with 227 beneficial side effects primarily concentrated in the circulatory system (31 distinct traits). Regarding PD motor progression, NUDT2 was associated with 93 beneficial side effects, predominantly within the circulatory system (15 traits). For PD composite progression, SH3BGRL3 conferred 67 beneficial side effects—the highest in this category—primarily related to digestive disorders. Finally, among candidate targets for PD onset, GGH showed 72 beneficial side effects, mainly affecting musculoskeletal conditions.  In contrast, the remaining 8 candidate targets displayed both beneficial and adverse side effects. Notably, targeting PD cognitive progression, GM2A was linked to 155 total side effects, comprising 56 beneficial and 99 adverse effects. For PD motor progression, EVA1C yielded 51 side effects, including 40 beneficial and 11 adverse effects. Lastly, for PD onset, FCGR2A was associated with 106 side effects, including 42 beneficial and 64 adverse effects. No significant side effects were detected among the candidate targets for PD composite progression |
|  | c) | If relevant, consider translating estimates of relative risk into absolute risk for a meaningful time period | / | / |
|  | d) | Consider plots to visualize results (e.g. forest plot, scatterplot of associations between genetic variants and outcome versus between genetic variants and exposure) | 15,16,19,23 | Fig 3，Fig4B，Fig 5B，Fig 7 |
| 12 | **Assessment of assumptions** |  |  |  |
|  | a) | Report the assessment of the validity of the assumptions | 7 | SMR is an extension of MR, and MR adheres to three core assumptions: (i) the relevance assumption, which requires a strong association between IVs and the exposure; (ii) the independence assumption, stating that IVs influence the outcome solely through the exposure; (iii) the exclusion restriction assumption, which dictates that IVs should not have a direct impact on the outcome. |
|  | b) | Report any additional statistics (e.g., assessments of heterogeneity across genetic variants, such as *I^2^*, Q statistic or E-value) | 7 | Unlike conventional two-sample MR, where two independent GWAS datasets are required to estimate the causal effect between traits, SMR combines pQTL and GWAS data and utilizes the Heterogeneity in Dependent Instruments (HEIDI) test. This approach offers more robust discrimination between pleiotropic and linkage effects, reduces potential biases due to LD, and lowers the large sample size requirements often seen in standard MR methods |
| 13 | **Sensitivity analyses and additional analyses** |  |  |  |
|  | a) | Report any sensitivity analyses to assess the robustness of the main results to violations of the assumptions | 7 | Unlike conventional two-sample MR, where two independent GWAS datasets are required to estimate the causal effect between traits, SMR combines pQTL and GWAS data and utilizes the Heterogeneity in Dependent Instruments (HEIDI) test. This approach offers more robust discrimination between pleiotropic and linkage effects, reduces potential biases due to LD, and lowers the large sample size requirements often seen in standard MR methods |
|  | b) | Report results from other sensitivity analyses or additional analyses | 7 | Unlike conventional two-sample MR, where two independent GWAS datasets are required to estimate the causal effect between traits, SMR combines pQTL and GWAS data and utilizes the Heterogeneity in Dependent Instruments (HEIDI) test^30^. This approach offers more robust discrimination between pleiotropic and linkage effects, reduces potential biases due to LD, and lowers the large sample size requirements often seen in standard MR methods |
|  | c) | Report any assessment of direction of causal relationship (e.g., bidirectional MR) | / | / |
|  | d) | When relevant, report and compare with estimates from non-MR analyses | / | / |
|  | e) | Consider additional plots to visualize results (e.g., leave-one-out analyses) | 15,16,19,23 | Fig 3，Fig4B，Fig 5B，Fig 7 |
|  | **DISCUSSION** |  |  |  |
| 14 | **Key results** | Summarize key results with reference to study objectives | 26 | Our study identified and validated latent plasma and brain targets for the onset and progression of PD, with the core means of an integrative PWAS, a method that has been demonstrated to be effective in such contexts. The study's findings, which are based on extensive pQTL data from plasma and brain tissues, accompanied by the utilization of comprehensive GWAS summary statistics. The culmination of our analysis resulted in the identification of 25 protein targets associated with PD, including onset and progression representing the trajectory. Furthermore, for the prioritized targets, we provided comprehensive insights into the therapeutic potential and safety profiles through PheW-MR analysis, cellular distribution-based clustering, PPI networks, and druggability assessments. |
| 15 | **Limitations** | Discuss limitations of the study, taking into account the validity of the IV assumptions, other sources of potential bias, and imprecision. Discuss both direction and magnitude of any potential bias and any efforts to address them | 13 | Despite the comprehensive nature of this proteome-wide association study, several limitations warrant consideration. First, our analyses do not encompass the entirety of the human proteome. Some proteins remained unmeasured and may also play pivotal roles in PD onset and progression, introducing the possibility of horizontal pleiotropy. Second, although our investigation incorporated both plasma and brain pQTL datasets, the absence of training sets in OTTERS for PWAS among brain proteins may reflect limitations in assay sensitivity and completeness of the entire study pipeline, partially weakening the convincement. Third, our cohort primarily includes individuals of Icelandic and European ancestries, where population homogeneity might reduce the generalizability of our findings to more diverse ethnic backgrounds, underscoring the need for replication efforts in multiethnic cohorts. Fourth, different proteomic platforms were employed (SOMAscan for plasma vs. mass spectrometry for brain), which may partially explain the minimal overlap of candidate targets across tissues. Harmonizing platform technologies in future studies could help identify additional shared targets. Fifth, the exclusion of EHBP1 from our downstream analyses due to missing expression data may have obscured its potential relevance in PD pathophysiology. Finally, the result of prioritized protein targets were computational hypotheses based on limited direct evidence, which is relatively primary and calls for experimental validation in the future. Addressing these limitations through broader proteomic profiling, larger and more diverse cohorts, and uniform assay platforms will be vital to refining our understanding of causal protein targets and their translational potential in PD. |
| 16 | **Interpretation** |  |  |  |
|  | a) | Meaning: Give a cautious overall interpretation of results in the context of their limitations and in comparison with other studies | 29.30 | Concerning the identified progression-related targets, ALKBH3, GLO1, IDO1, SERPINA3, SORD, TPST1, GM2A, MICB, SH3BGRL3, and TGFBI, we found them to be significantly associated with cognitive decline in the plasma of PD patients. They are involved in diverse biological processes: expression and glycation damage of GLO1 was demonstrated to be induced by alpha-synuclein ablation, contributing to the development of PD40; IDO1 inhibition improves motor dysfunction and provides neuroprotection in PD mice; MICB and TPST1 are novel targets identified for PD, may shape neuroinflammatory processes by modulating microglial activation in PD and mediate sulfation of key neuronal proteins modulating intracellular signaling pathways, thereby influencing dopaminergic neuron survival and accelerate the progression; Additionally, targets such as NUDT2, PLA2G12B, EVA1C, and MATN3 were associated with motor progression, highlighting potential targets for mitigating motor dysfunction in PD. For instance, NUDT2, involved in nucleotide metabolism, and PLAG12B in PLA2 (phospholipase A2) superfamily was suggestively associated with PD, influencing neuronal membrane integrity and essential signal transduction pathways for motor function. The identification of these targets underscores the complex interplay between metabolic and inflammatory pathways in PD motor symptoms. Furthermore, SH3BGRL3 was identified as an intriguing target, demonstrating a dual role by being causally linked to both cognitive and composite progression phenotypes, albeit with contrasting directions of effect. While there is no direct evidence, this duality suggests that SH3BGRL3 may regulate multiple pathways that differentially affect various aspects of disease progression, such as influencing PD by stabilizing synaptic architecture and acting as a redox sensor, thereby protecting against α-synuclein-induced synaptic deficits and adjusting dysregulated intracellular signaling cascades. Apart from that, previously hinted by an MR study, GPNMB stood out as a pivotal target showing a causal relationship with increased risk of PD onset in both plasma and brain tissues. The consistent association of GPNMB across different tissues highlights its potential as a robust biomarker for early PD detection and as a promising therapeutic target to delay disease onset. |
|  | b) | Mechanism: Discuss underlying biological mechanisms that could drive a potential causal relationship between the investigated exposure and the outcome, and whether the gene-environment equivalence assumption is reasonable. Use causal language carefully, clarifying that IV estimates may provide causal effects only under certain assumptions | 26,27 | Concerning the identified progression-related targets, ALKBH3, GLO1, IDO1, SERPINA3, SORD, TPST1, GM2A, MICB, SH3BGRL3, and TGFBI, we found them to be significantly associated with cognitive decline in the plasma of PD patients. They are involved in diverse biological processes: expression and glycation damage of GLO1 was demonstrated to be induced by alpha-synuclein ablation, contributing to the development of PD40; IDO1 inhibition improves motor dysfunction and provides neuroprotection in PD mice; MICB and TPST1 are novel targets identified for PD, may shape neuroinflammatory processes by modulating microglial activation in PD and mediate sulfation of key neuronal proteins modulating intracellular signaling pathways, thereby influencing dopaminergic neuron survival and accelerate the progression; Additionally, targets such as NUDT2, PLA2G12B, EVA1C, and MATN3 were associated with motor progression, highlighting potential targets for mitigating motor dysfunction in PD. For instance, NUDT2, involved in nucleotide metabolism, and PLAG12B in PLA2 (phospholipase A2) superfamily was suggestively associated with PD, influencing neuronal membrane integrity and essential signal transduction pathways for motor function45. The identification of these targets underscores the complex interplay between metabolic and inflammatory pathways in PD motor symptoms. Furthermore, SH3BGRL3 was identified as an intriguing target, demonstrating a dual role by being causally linked to both cognitive and composite progression phenotypes, albeit with contrasting directions of effect. While there is no direct evidence, this duality suggests that SH3BGRL3 may regulate multiple pathways that differentially affect various aspects of disease progression, such as influencing PD by stabilizing synaptic architecture and acting as a redox sensor, thereby protecting against α-synuclein-induced synaptic deficits and adjusting dysregulated intracellular signaling cascades. Apart from that, previously hinted by an MR study, GPNMB stood out as a pivotal target showing a causal relationship with increased risk of PD onset in both plasma and brain tissues. The consistent association of GPNMB across different tissues highlights its potential as a robust biomarker for early PD detection and as a promising therapeutic target to delay disease onset. |
|  | c) | Clinical relevance: Discuss whether the results have clinical or public policy relevance, and to what extent they inform effect sizes of possible interventions | 29 | Our study is underpinned by several notable strengths that collectively enhance its scientific rigor and potential impact. Firstly, our research represents the first known PWAS utilizing the OTTERS method and large-scale summary-level pQTL data from deCODE to investigate both the onset and progression of PD. In contrast to previous PWAS studies that employed small-sample pQTL data, we leveraged the OTTERS framework with extensive summary-level pQTL data from deCODE. This methodological approach significantly increases statistical power, enabling the identification of a greater number of critical proteins, particularly those previously undiscovered. Consequently, this not only deepens our understanding of the mechanisms driving PD progression but also provides additional potential targets for developing disease-modifying therapeutic strategies. Furthermore, the robustness of our findings is reinforced through rigorous validation methodologies, including SMR, colocalization analyses, and bidirectional MR. These approaches collectively ensure high confidence in the causal relationships between proteins and PD phenotypes. Additionally, our comprehensive assessment of potential side effects via PheW-MR offers critical insights into the safety profiles of candidate targets, thereby informing the development of safer therapeutic interventions. The identification of existing drug targets among the candidates also facilitates drug repurposing, potentially accelerating the translation of our findings into clinical applications and enhancing the feasibility of novel therapeutic strategies. |
| 17 | **Generalizability** | Discuss the generalizability of the study results (a) to other populations, (b) across other exposure periods/timings, and (c) across other levels of exposure | 30,31 | First, our analyses do not encompass the entirety of the human proteome. Some proteins remained unmeasured and may also play pivotal roles in PD onset and progression, introducing the possibility of horizontal pleiotropy. Second, although our investigation incorporated both plasma and brain pQTL datasets, the absence of training sets in OTTERS for PWAS among brain proteins may reflect limitations in assay sensitivity and completeness of the entire study pipeline, partially weakening the convincement. Third, our cohort primarily includes individuals of Icelandic and European ancestries, where population homogeneity might reduce the generalizability of our findings to more diverse ethnic backgrounds, underscoring the need for replication efforts in multiethnic cohorts. |
|  | **OTHER INFORMATION** |  |  |  |
| 18 | **Funding** | Describe sources of funding and the role of funders in the present study and, if applicable, sources of funding for the databases and original study or studies on which the present study is based | 31 | This work was supported by National Natural Science Foundation of China (Grants 81671068, 81873727, 82171196, 82304990) and the China Postdoctoral Science Foundation (Grant No. 2023M732380). |
| 19 | **Data and data sharing** | Provide the data used to perform all analyses or report where and how the data can be accessed, and reference these sources in the article. Provide the statistical code needed to reproduce the results in the article, or report whether the code is publicly accessible and if so, where | 30 | Each cohort included in this study has been conducted using published studies and consortia providing publicly available summary statistics. All original studies have received ethical approval and agreed to participate, and summary-level data were provided for analysis.  Publicly available datasets were analyzed in this study. This data can be found on deCODE Health study(https://www.decode.com/summarydata/), ROS/MAP study(https://www.synapse.org/#!Synapse:syn23627957), GWAS Catelog(https://www.ebi.ac.uk/gwas/home) FinnGen consortium(https://www.finngen.fi/fi), Allen Brain Cell Atlas Public Dataset(https://allen-brain-cell-atlas.s3.us-west-2.amazonaws.com/index.html). |
| 20 | **Conflicts of Interest** | All authors should declare all potential conflicts of interest | 30 | The authors declare that there is no conflict of interest. |

This checklist is copyrighted by the Equator Network under the Creative Commons Attribution 3.0 Unported (CC BY 3.0) license.

1. Skrivankova VW, Richmond RC, Woolf BAR, Yarmolinsky J, Davies NM, Swanson SA, et al. Strengthening the Reporting of Observational Studies in Epidemiology using Mendelian Randomization (STROBE-MR) Statement. JAMA. 2021;under review.

2. Skrivankova VW, Richmond RC, Woolf BAR, Davies NM, Swanson SA, VanderWeele TJ, et al. Strengthening the Reporting of Observational Studies in Epidemiology using Mendelian Randomisation (STROBE-MR): Explanation and Elaboration. BMJ. 2021;375:n2233.
